# Targeting IL-1α Sensitizes HNSCC to PDT by Reversing Hypoxia and NF-κB-Driven Oxidative Stress Resistance

**DOI:** 10.7150/ijbs.131696

**Published:** 2026-06-04

**Authors:** Zhiyin Li, Yikang Ji, Xinran Zhao, Hexin Ma, Wanling Chen, Zijie Zhou, Xu Wang, Lingyue Shen, Lingyan Zheng

**Affiliations:** 1 Department of Oral Surgery, Shanghai Ninth People's Hospital, Shanghai Jiao Tong University, School of Medicine; College of Stomatology, Shanghai Jiao Tong University; National Center for Stomatology; National Clinical Research Center for Oral Diseases; Shanghai Key Laboratory of Stomatology; Shanghai Research Institute of Stomatology; Shanghai 200011, P.R. China.; 2 Department of Oral and Maxillofacial-Head and Neck Oncology, Shanghai Ninth People's Hospital, Shanghai Jiao Tong University School of Medicine; College of Stomatology, Shanghai Jiao Tong University; National Center for Stomatology; National Clinical Research Center for Oral Diseases; Shanghai Key Laboratory of Stomatology; Shanghai Research Institute of Stomatology; Shanghai Center of Head and Neck Oncology Clinical and Translational Science; Shanghai 200011, P.R. China.; 3 Department of Oral Pathology, Shanghai Ninth People's Hospital, Shanghai Jiao Tong University, School of Medicine; College of Stomatology, Shanghai Jiao Tong University; National Center for Stomatology; National Clinical Research Center for Oral Diseases; Shanghai Key Laboratory of Stomatology; Shanghai 200011, P.R. China.; 4 Department of Laser and Aesthetic Medicine, Shanghai Ninth People's Hospital, Shanghai Jiao Tong University School of Medicine, Shanghai 200011, P.R. China.

**Keywords:** head and neck squamous cell carcinoma, photodynamic therapy, IL-1α, oxidative resistance, spatial transcriptomics, patient-derived organoids

## Abstract

Photodynamic therapy (PDT) provides non-invasive precision for superficial lesions but achieves suboptimal responses in hypoxic tumors such as head and neck squamous cell carcinoma (HNSCC). Spatially heterogeneous resistance mechanisms pose a major translational constraint for extending PDT applicability. To overcome this limitation, actionable targets were mapped within tumor spatial heterogeneity, circumventing conventional nanomaterial-based hypoxia-reversal strategies with inherent design complexity and protracted translation timelines. Spatial transcriptomic profiling of HNSCC specimens revealed pronounced molecular gradients along hypoxic regions, pinpointing *IL1A* as the most significantly upregulated transcript in hypoxic niches, which directly correlates with adverse clinical outcomes. PDT further amplified IL-1α expression, establishing a self-reinforcing resistance loop. Functional analyses confirmed that hypoxic tumor-derived IL-1α activates the NF-κB pathway to confer resistance against PDT-induced oxidative stress. Critically, the selective IL-1R1 antagonist AF12198 disrupted this resistance axis, significantly enhancing PDT efficacy across cellular models and patient-derived organoids (PDOs). Pharmacological blockade of the IL-1α/IL-1R1/NF-κB axis represents a clinically actionable strategy against intrinsic PDT resistance. By leveraging spatial heterogeneity to identify IL-1α as a druggable target, this study provides robust preclinical support for repurposing clinical IL-1 inhibitors to enhance PDT efficacy.

## Introduction

Photodynamic therapy (PDT) is a promising minimally invasive cancer treatment 1. This modality utilizes a photosensitizer (PS) activated by specific light to generate cytotoxic reactive oxygen species (ROS), enabling selective tumor cell elimination 2, 3. Its non-invasiveness and precise spatiotemporal control support its clinical investigation for head and neck squamous cell carcinoma (HNSCC) and other cancers 4-6. For this study, we selected hematoporphyrin monomethyl ether (HMME), an FDA-approved PS clinically used for port-wine stain PDT 7, to establish HNSCC models. However, despite efficacy against superficial lesions, treatment resistance severely compromises PDT's clinical utility 8, 9.

The hypoxic microenvironment is a hallmark of solid tumors that critically compromises PDT efficacy. It limits the generation of cytotoxic ROS, which are essential mediators of PDT-induced cell death 10, 11. This challenge is exacerbated by the inherent paradox of PDT: its oxygen-dependent type II photochemical reaction consumes molecular oxygen within already hypoxic tumors. This creates a self-limiting cycle that intensifies hypoxia and reduces therapeutic efficacy 12, 13. Concurrently, tumor cells activate adaptive responses to oxidative stress. Key transcription factors, such as hypoxia-inducible factor-1α (HIF-1α) and nuclear factor erythroid 2-related factor 2 (NRF2), orchestrate the upregulation of antioxidant molecules and enzymes, including glutathione peroxidase 4 (GPX4) and superoxide dismutase. These defenses mitigate ROS-induced damage while promoting pro-survival signaling and angiogenesis, collectively fostering a resistant phenotype 14-16. The convergence of extrinsic oxygen limitation and intrinsic adaptive resistance thus constitutes a major mechanistic basis for PDT failure.

Conventional strategies aimed at reversing tumor hypoxia in PDT primarily rely on nanomaterial-based approaches 17. These can be broadly include: 1) Oxygen conservation via mitochondrial inhibitors or “oxygen economizers” to suppress cellular respiration 18, 19; 2) Oxygen generation through nanozymes with catalase-like activity that decompose elevated levels of hydrogen peroxide (H_2_O_2_) in tumor microenvironment, or glutathione (GSH) depletion to minimize ROS scavenging 13, 20; 3) Oxygen deliverly using nanocarriers such as biomimetic erythrocyte vesicles or perfluorocarbon particles to increase oxygen level to tumors 12, 21. Despite preclinical promise, these nano-strategies face significant translational hurdles, including complex fabrication, scalability issues, potential long-term toxicity, and high development costs, which lead to lengthy clinical translation timelines. More critically, tumor cells' remarkable plasticity enables compensatory pathway activation that bypasses the therapeutic pressure induced by oxygen modulation, driving adaptive resistance and limiting durable efficacy 22, 23. Given these translational barriers, we must shift the therapeutic paradigm to pivot from complex material engineering toward targeting molecular vulnerabilities within the tumor's spatial ecosystem. Identifying key druggable resistance drivers specifically in hypoxic niches offers a clinically viable alternative for developing more clinically tractable PDT-sensitizing strategies. Direct disruption of core signaling nodes used by tumors to survive PDT stress could circumvent nanomaterial limitations and improve therapeutic outcomes.

In this study, we aim to pursue a target-centric strategy by mapping molecular vulnerabilities within tumor spatial ecosystems. Through tripartite profiling of HMME-mediated PDT proteomes, hypoxic versus normoxic niche transcriptomes (spatial transcriptomics), and oxidative stress-responsive secretomes, we discovered interleukin-1 alpha (IL-1α) as a hypoxia-induced mediator in therapy-resistant niches correlated with poor prognosis. Crucially, PDT amplified IL-1α expression, activating the IL-1R1/pIRAK4/NF-κB antioxidant axis. Pharmacological blockade of this pathway using IL-1R1 antagonist AF12198 significantly restored PDT efficacy across cellular and patient-derived organoid models (PDOs). Our results establish a spatially guided approach to overcome intrinsic PDT resistance, translating target discovery from patient tumor ecosystems into a clinically actionable therapeutic paradigm that circumvents the limitations of nanomaterial-based hypoxia modulation.

## Materials and Methods

### Human tissue specimens

Primary tumor tissues and matched adjacent normal tissues were obtained from HNSCC patients undergoing surgical resection at the Department of Oral and Maxillofacial-Head and Neck Oncology, Shanghai Ninth People's Hospital, Shanghai Jiao Tong University School of Medicine.

### Animals

C3He and BALB/c nude mice (6 weeks old) were housed in the specific pathogen-free (SPF) animal facility at Shanghai Ninth People's Hospital, Shanghai Jiao Tong University School of Medicine. All animals were maintained under standard conditions: room temperature (22-25°C), ad libitum access to food and water, and a 12 h light/dark cycle. After quarantine, animals were acclimatized for one week before experiments.

### Cell culture

The human HNSCC cell lines HN6, HN30, CAL27, SCC9, and SCC25, and the murine HNSCC cell line SCC7 were utilized. HN6, HN30, CAL27, SCC9, and SCC25 originate from human oral squamous cell carcinoma; SCC7 derives from C3He mouse tumors. HN6, HN30, CAL27, and SCC7 were cultured in high-glucose Dulbecco's Modified Eagle Medium (DMEM; TBD, China) supplemented with 10% fetal bovine serum (FBS; TBD, China) and 1% penicillin-streptomycin (PS; Beyotime, China). SCC9 and SCC25 were cultured in DMEM/F12 medium (TBD, China) supplemented with 10% FBS and 1% PS. All cell lines were maintained at 37 °C in a humidified atmosphere containing 5% CO_2_. Cells were incubated under either normoxic (21% O_2_) or hypoxic (1% O_2_) conditions as required for specific experiments.

### Organoids culture

Tumor tissues from HNSCC patients were minced into approximately 3-mm^3^ fragments and washed with DMEM (Gibco, USA) containing 5% PS. Tissue fragments were enzymatically dissociated into single cells using DMEM containing collagenase II (Sigma-Aldrich, USA) and DNase I (Applichem, Germany) at 37 °C for 2 h with gentle agitation. The dissociated cells were diluted in complete medium and sequentially filtered through 100 μm and 70 μm strainers. After dissociation at 200 g for 3 min, cells were counted and resuspended in Matrigel basement membrane matrix (R&D Systems, USA). The cell-Matrigel suspension was plated as dome-shaped droplets in a preheated (37 °C) 48-well plate and overlaid with culture medium. Cultures were maintained at 37 °C in a humidified 5% CO_2_ incubator. Organoid formation was monitored starting from day 7, with medium changes every 3 days. Organoids were passaged every two weeks at a 1:5 to 1:6 split ratio. For cryopreservation, organoids were recovered from Matrigel and frozen in serum-free cell cryopreservation medium (NCM Biotech, China).

### siRNA interference

HN6 and SCC7 cells were seeded in 6-well plates and cultured in DMEM supplemented with 10% FBS. Upon reaching approximately 60% confluence, cells were washed and incubated with serum-free DMEM for transfection. Transfection was performed using Lipofectamine 3000 reagent (Thermo Fisher Scientific, USA) according to the manufacturer's protocol. Cells were transfected with 20 nM siRNA (designed and synthesized by Genomeditech, China) for 6 h. Following transfection, the medium was replaced with DMEM containing 10% FBS. The siRNA sequences are provided in the Supplementary Table 1.

### Lentiviral transduction

HN6 and HN30 cells were seeded in 24-well plates at a density of 5×10^4^ cells/well in 0.5 mL DMEM supplemented with 10% FBS and cultured overnight. Lentivirus dilution medium was prepared containing Polybrene (5 μg/mL) according to the manufacturer's protocol. Lentiviral particles (20-100 μL of primary stock) were added to this dilution medium. The diluted lentivirus mixture was then added to the cells. Control groups (untreated and negative control lentivirus) were established concurrently. After 24 h of incubation, the medium was replaced with fresh complete growth medium (DMEM with 10% FBS) and cultured for an additional 24 h. Transduced cells were selected using complete medium containing 2 μg/mL puromycin. Resistant cell populations were expanded in 10-cm dishes for subsequent experiments. Lentiviruses expressing specific shRNAs were designed and synthesized by Genomeditech (China). The shRNA sequences are provided in the Supplementary Table 2.

### Intracellular uptake assay

HN6, HN30, CAL27, SCC9, SCC25, and SCC7 cells were seeded at a density of 1×10^4^ cells per well in 4-chamber glass-bottom dishes. Cells were treated with HMME (5 μg/mL, Shanghai Fudanzhangjiang Bio-Pharmaceutical, China) for either 0 min (untreated control) or 3 h. Following treatment, cells were washed, and nuclei were counterstained with DAPI (5 min at room temperature). Cellular uptake of HMME was visualized using a confocal laser scanning microscope (CLSM; LSM 900, ZEISS, USA). HMME fluorescence was detected using excitation/emission wavelengths of 380 nm/631 nm, and DAPI was detected using excitation/emission wavelengths of 364 nm/454 nm.

### CCK-8 assay

To assess cell viability following HMME-mediated PDT and determine the IC_50_, HN6, HN30, CAL27, SCC9, SCC25, and SCC7 cells were seeded in 96-well plates at 5,000 cells/well and cultured until 80-90% confluent. After incubation with HMME at concentrations of 0, 0.01, 0.1, 1, 2, 5, 10, or 20 μg/mL for 3 h, cells were irradiated (532 nm laser, 100 mW/cm^2^, 3 min) and then cultured for an additional 24 h. Culture medium was replaced with 100 μL of fresh serum-free medium containing 10% (v/v) CCK-8 reagent (Cellcook, China). After incubating at 37°C for 2 h, absorbance was measured at 450 nm using a microplate reader (SpectraMax i3, Molecular Devices, USA). Relative cell viability (%) was calculated as follows: Relative Viability (%)=[(As - Ab)/(Ac - Ab)]×100. Where: As = Absorbance of sample well (cells+treatment); Ac = Absorbance of control well (cells+medium only); Ab = Absorbance of background well (medium only, no cells).

To evaluate the effect of *IL1A* knockdown on PDT sensitivity, lentivirus-transduced HN6 and HN30 cells expressing Sh-*IL1A* or control shRNA were seeded in 96-well plates (5,000 cells/well) until 80-90% confluent. After incubating with HMME (HN6: 7 μg/mL; HN30: 5 μg/mL) for 3 h, cells were irradiated (532 nm laser, 100 mW/cm^2^, 3 min) and cultured for varying post-irradiation time periods. Cell viability was determined using the CCK-8 assay as described above.

To assess the impact of IL-1α signaling blockade on PDT efficacy, HN6, HN30, CAL27, SCC9, SCC25, and SCC7 cells were seeded in 96-well plates (5,000 cells/well) until 80-90% confluent. Cells were treated for 3 h with one of the following: HMME alone (HN6: 7 μg/mL; other cell lines: 5 μg/mL); HMME and recombinant IL-1 receptor antagonist protein (IL-1RN; 200 ng/mL; MedChemExpress, China); HMME and AF12198 (500 nM; MedChemExpress, China). Following treatment, the non-irradiated control group cells were cultured for a total of 32 h (3 h treatment and 29 h incubation). While the PDT group cells were irradiated (532 nm laser, 100 mW/cm^2^, 3 min) immediately after the 3 h treatment, and cultured for an additional 29 h. Cell viability was determined using the CCK-8 assay as described above.

### Western blot assay

Western blotting was performed to analyze protein expression in the following experimental systems: 1) Expression of pre-IL-1α in HN6 cells under normoxic and hypoxic conditions, and tumor tissues versus adjacent normal tissues from HNSCC patients. 2) Expression of pre-IL-1α transduced with sh-*IL1A* lentivirus versus sh-Scramble control in HN6 and HN30 cells, and transfected with si-*IL1A* versus Scramble siRNA in HN6 and SCC7 cells. 3) Expression of IL-1α and pre-IL-1α in CAL27, HN6, and HN30 cells following HMME-mediated PDT (varying laser irradiation times and post-treatment durations). 4) Expression of NRF2, IL-1α, and pre-IL-1α in si-*NRF2*-transfected HN6 cells after HMME-PDT under normoxia and hypoxia. 5) Expression of phospho-T345-IRAK4, phospho-NF-κB p65, total IRAK4, and total p65 levels post-PDT treatment and supply recombinant IL-1α protein.

Cells were seeded in 6-well plates at a density of 3×10^5^ cells/well and cultured until 80-90% confluent. After receiving the specified treatments, cells were lysed on ice using RIPA lysis buffer (NCM Biotech, China) supplemented with protease and phosphatase inhibitors. Total protein concentration was determined using the BCA Protein Assay Kit (Thermo Fisher Scientific, USA). Protein lysates were mixed with 5× SDS-PAGE loading buffer (final 1×), denatured by boiling at 100 °C for 15 min, and resolved by electrophoresis on SDS-polyacrylamide gels (Genshare Biological, China). Separated proteins were electrophoretically transferred onto 0.22 μm polyvinylidene fluoride (PVDF) membranes (Merck Millipore, USA). Membranes were blocked with 5% (w/v) non-fat dry milk in Tris-buffered saline containing 0.1% Tween-20 (TBST) for 1 h at room temperature. After blocking, membranes were washed three times (5 min each) with TBST and incubated with primary antibodies overnight at 4 °C. Membranes were washed three times (10 min each) with TBST and incubated with horseradish peroxidase (HRP)-conjugated goat anti-rabbit IgG secondary antibody (Beyotime, China; A0208, 1:2000) for 1 h at room temperature. After washing extensively with TBST (3×10 min), protein bands were visualized using Immobilon Forte Western HRP Substrate (Merck Millipore, USA) and detected using an Amersham Imager 600 system (General Electric Company, USA). The primary antibodies used were *anti*-IL-1α (Abclonal, China; A1316, 1:1000), *anti*-NRF2 (Abclonal, China; A3577, 1:1000), *anti*-IRAK4 (Proteintech, China; 18221-1-AP, 1:3000), *anti*-pThr-IRAK4 (Bioss, China; bs-10208R, 1:2000), *anti*-NF-κB p65/RelA (Abclonal, China; A19653, 1:5000), *anti*-Phospho-NF-κB p65/RelA (Ser536) (Abclonal, China; AP0124, 1:2000), and *anti*-β-actin (Beyotime, China; AF5003, 1:1000; loading control).

### Quantitative real-time PCR (qPCR)

qPCR was performed to assess the knockdown efficiency of *IL1A*-targeting siRNA in HN6 and SCC7 cells. Total RNA was isolated using the RNA-Quick Purification Kit (Yishan Biotechnology, China) according to the manufacturer's instructions. RNA concentration and purity were determined spectrophotometrically. First-strand cDNA was synthesized from 1 μg total RNA using the PrimeScript RT Reagent Kit (Takara Bio, Japan) with oligo (dT) primers. qPCR reactions were carried out using Hieff UNICON® qPCR SYBR Green Master Mix (Yeasen Biotechnology, China) on a QuantStudio 6 Flex Real-Time PCR System (Applied Biosystems, USA). Each 20 μL reaction contained 10 μL Master Mix, 0.25 μM of each primer, and 2 μL diluted cDNA template. The thermal cycling conditions were as follows: initial denaturation at 95 °C for 5 min; 40 cycles of 95 °C for 10 s and 60 °C for 30 s; followed by a melt curve analysis. The amplified PCR products were quantified and normalized using β-Actin as a reference gene. Primer sequences are provided in the Supplementary Table 3.

### ROS detection assay

Intracellular ROS production during HMME-mediated PDT was assessed under the following conditions: siRNA-mediated knockdown of *IL1A*, blockade of IL-1α signaling using IL-1RN (200 ng/mL) or AF12198 (500 nM), and exogenous supplementation with recombinant IL-1α protein (200 ng/mL; all reagents from MedChemExpress, China). HN6 and SCC7 cells were treated with HMME for 3 h, followed by irradiation for 3 min (532 nm laser, 100 mW/cm^2^), and continued to incubate for an additional 8 h. After washing twice with pre-warmed PBS, cells were incubated with 10 μM 2',7'-dichlorodihydrofluorescein diacetate (DCFH-DA; Beyotime, China) in serum-free medium at 37 °C in the dark for 20 min. ROS generation was visualized immediately using CLSM (LSM 900, ZEISS, USA). Fluorescence was detected using excitation/emission wavelengths of 488 nm/525 nm.

### Oxygen detection assay

HN6 cells were pre-incubated under hypoxic conditions (1% O_2_) for 12 h in the presence of the oxygen-sensitive probe [Ru(dpp)_3_]Cl_2_ (Shanghai Maokang Biotech, China). The PDT group cells were incubated with HMME (7 μg/mL) for 3 h, irradiated for 3 min (532 nm laser, 100 mW/cm^2^), and further incubated for 3 h; the control group cells were incubated with HMME (7 μg/mL) for 6 h without laser irradiation. Following treatment, the intracellular oxygen levels were immediately visualized using CLSM (LSM 900, ZEISS, USA). Probe signal was detected using excitation/emission wavelengths of 488 nm/610 nm.

### Enzyme-linked immunosorbent assay (ELISA)

To quantify secreted IL-1α in response to HMME-mediated PDT, HN6 and SCC7 cells were seeded at 3×10^5^ cells/well in 6-well plates and cultured until 80-90% confluent. The control group cells were incubated with HMME for 9 h, and the PDT group cells were incubated for 3 h, followed by irradiation for 3 min (532 nm laser, 100 mW/cm^2^), and then cultured for an additional 6 h. After treatment, cell culture supernatants were collected and centrifuged at 1,000 g for 20 min at 4 °C to remove debris. Levels of IL-1α in the clarified supernatants were quantified by the human IL-1α ELISA kit (Hengyuan, China) for HN6 supernatants and the mouse IL-1α ELISA kit (Hengyuan, China) for SCC7 supernatants. Assays were performed strictly according to the manufacturers' protocols. Absorbance was measured at 450 nm using a microplate reader (SpectraMax i3, Molecular Devices, USA) after a 30-min incubation with the stop solution.

### Proteomics

HN6 cells were cultured in 10-cm dishes until 90% confluent. After discarding the medium, cells were washed once with PBS (TBD, China). The control group was incubated with 10 μg/mL HMME for 11 h, and the PDT group was incubated with 10 μg/mL HMME for 3 h, irradiated for 3 min (532 nm laser, 100 mW/cm^2^), and further incubated for 8 h. After treatment, dishes were inverted on absorbent paper to remove residual medium. Cells were washed three times with ice-cold PBS (4 °C; 1 min/wash with gentle shaking) and transferred to pre-chilled tubes. After centrifugation (1,000 g, 5 min, 4 °C) and flash-frozen in liquid nitrogen, cell pellets were stored at -80 °C. Protein was extracted and digested. LC-MS/MS analysis was performed on a timsTOF Pro mass spectrometer (Bruker) that was coupled to Nanoelute (Bruker). The MS raw data for each sample were combined and searched using the MaxQuant 1.6.14 software for identification and quantitation analysis. The LC-MS/MS analysis was performed by Shanghai Applied Protein Technology Co., Ltd.

### RNA sequencing (RNA-seq)

HN6 cells were seeded in 10-cm dishes and cultured to 90% confluency. After discarding the medium, cells were washed once with PBS. The control group was incubated with 10 μg/mL HMME; the treatment group was incubated with 10 μg/mL HMME and 500 nM AF12198. Both groups were treated for 3 h, irradiated for 3 min (532 nm laser, 100 mW/cm^2^), and further incubated for 8 h. After treatment, the medium was discarded, and the dishes were inverted on absorbent paper to remove residual liquid. Cells were washed three times with ice-cold PBS (1 min/wash) to completely remove medium components. Total RNA was extracted using TRIzol^TM^ Reagent (Invitrogen, USA) according to the manufacturer's protocol. RNA-seq assay was performed by OEBiotech China. Total RNA was extracted, and a cDNA library was prepared according to the standard Illumina RNA-seq instructions. Hisat2 was selected as the mapping tool, and FeatureCounts v1.5.0-p3 was used to count the read numbers mapped to each gene.

### Immunofluorescence assay

For cell immunofluorescence assays, the HN6 cells were treated with HMME-mediated PDT under normoxic or hypoxic conditions to confirm NRF2 activation and nuclear translocation. The HN6 and HN30 cells treated with HMME-mediated PDT, accompanied by sh-*IL1A*, si-*IL1A*, or AF12198, to reveal phospho-NF-κB p65 activation in IL-1α-blocked models. After incubating for 3 h and irradiating for 3 min, the cells were further incubated for 6 h and fixed with 4% paraformaldehyde for 15 min at room temperature. Cells were penetrated with 0.5% Triton X-100 for 10 min and blocked with 1% BSA (Beyotime, China) for 1 h. After incubating with primary antibodies at 4 ℃ overnight, the samples were stained with Alexa Fluor 647-conjugated anti-rabbit IgG secondary antibodies (Cell Signaling Technology, 4414S; 1:200) for 1 h at room temperature. Nuclei were visualized by DAPI staining for 5 min. The primary antibodies used were *anti*-NRF2 (Abclonal, China; A3577; 1:200) and *anti*-Phospho-NF-κB p65 (Ser536) (Abclonal, China; AP0124; 1:200). Pictures were taken by CLSM (LSM900, ZEISS, USA).

For tissue immunofluorescence assays, paraffin-embedded tissue sections (3 μm thickness) were deparaffinized in xylene and rehydrated through a graded ethanol series. Antigen retrieval was performed by incubating sections in citrate buffer at 95 °C for 20 min. After cooling to room temperature, sections were washed three times with TBST and then permeabilized with 0.5% Triton X-100 in PBS for 15 min. After blocking with 3% goat serum albumin (Beyotime, China) for 1 h, the primary antibodies were applied and incubated overnight at 4 °C. After TBST washes, sections were incubated with secondary antibodies for 1 h at room temperature. Nuclei were counterstained with DAPI for 10 min. The primary antibodies used were *anti*-IL-1α (Abclonal, China; A1316; 1:200), *anti*-HIF-1α (Cell Signaling Technology, USA; 36169T; 1:400), and *anti*-Pan-CK (Maxim Biotechnology, China, Kit-0009; 1:200). The secondary antibodies used were Goat anti-Rabbit IgG-Alexa Fluor 488 (for IL-1α), Goat anti-Rabbit IgG-Alexa Fluor 647 (for HIF-1α), and Goat anti-Mouse IgG-Alexa Fluor 594 (for Pan-CK). Images were obtained with ZEISS LSM880 scanning confocal microscope (ZEISS, Germany) and analyzed by using the ImageJ software for the colocalization of HIF-1α and IL-1α*.*

### Live/dead staining assay

P3-HNSCC-derived organoids were used when organoid structures occupied 70-80% of the Matrigel dome. The culture medium was replaced with fresh medium containing 150 μg/mL HMME and 100 μM AF12198. After 3 h incubation at 37 °C, PDT subgroups then received laser irradiation (5 min) followed by 24 h culture, while control subgroups continued culture without irradiation. Then, PDOs were stained with Calcein-AM/PI Viability Kit (Beyotime, China; C2015S) working solution (1:1 ratio) for 30 min at 37 °C protected from light. Brightfield and fluorescence images were acquired by using BioTek Cytation 5 (BioTek, USA) with Calcein (live cells: Ex/Em=494/517 nm) and PI (dead cells: Ex/Em=535/617 nm).

### 3D cell viability assay

P3-HNSCC-derived organoids were planted in an opaque 96-well plate when organoid structures occupied 70-80% of the Matrigel dome. The PDOs were treated in the same way as mentioned above. Discard the old culture medium, and add 100 μL of detection CellTiter-Glo® 3D reagent (Promega Biotech, China, G9863) equilibrated to room temperature to each well. Shake thoroughly for 5 min to ensure complete cell lysis. Subsequently, incubate the plate at room temperature for an additional 25 min to stabilize the luminescence signal, and measure the chemiluminescence using a microplate reader (SpectraMax i3, Molecular Devices, USA).

### Establishment and treatment of the mouse subcutaneous xenograft model

To verify HMME-PDT-induced IL-1α expression *in vivo*, female C3He mice (6-week-old) were subcutaneously injected with SCC7 cells (5×10^5^ cells in 100 μL PBS) into the right flank. The mice were randomized into two groups (n = 5) when tumors reached 200 mm^3^. The PDT group was administered with HMME (1 mg/kg) by tail vein injection. After 3 h, the mice were irradiated for 3 min (532 nm laser, 160 mW/cm^2^) under anesthesia with 1 % pentobarbital sodium. Body weight and tumor volume (Volume = 0.5×Length×Width^2^) were monitored every 2 days. Mice were euthanized by CO_2_ asphyxiation if tumor volume exceeded 1000 mm^3^.

To assess *IL1A* knockdown efficacy in PDT, male BALB/c nude mice (6-week-old) received subcutaneous injections of sh-Scramble and sh-*IL1A* HN6 cells (1×10^6^ cells in 100 μL PBS). On Day 5 post-inoculation, mice were randomized into four groups (n = 4) as sh-Scramble NC, sh-Scramble PDT, sh-*IL1A* (no PDT), and sh-*IL1A* PDT. On Days 16 and 29, PDT groups received HMME (1 mg/kg, i.v.) followed by laser irradiation (identical parameters). Control groups received saline. Tumor growth was tracked until the endpoint as above.

To evaluate AF12198 enhancement of PDT, male BALB/c nude mice (6-week-old) received subcutaneous injections of HN6 cells (1×10^6^ cells in 100 μL PBS). On Day 7 post-inoculation, mice were randomized into four groups (n = 4) as Control (Saline), AF12198 (3.35 mg/kg, i.v.), PDT (HMME 1 mg/kg, i.v.), and HMME combined with AF12198. The drug was injected on days 11 and 26, and the indicated groups were followed by laser irradiation (identical parameters). All procedures followed identical monitoring and endpoint criteria.

### H&E staining and immunohistochemistry (IHC)

For H&E staining, paraffin-embedded tissue sections (3 μm thickness) were deparaffinized in xylene and rehydrated through a graded ethanol series. Nuclei were stained with Mayer's hematoxylin (Beyotime, China) for 8 min, followed by differentiation in 1% acid ethanol and bluing in Scott's tap water substitute. Cytoplasm was counterstained with 0.5% eosin Y for 1 min. Sections were dehydrated through graded ethanol, cleared in xylene, and mounted with neutral balsam.

For IHC staining, sections underwent deparaffinization and rehydration as above, followed by heat-induced antigen retrieval in citrate buffer (pH 6.0) at 95 °C for 20 min. Endogenous peroxidase activity was blocked with 3% H_2_O_2_ for 15 min. After blocking with 3% goat serum (Beyotime, China) for 1 h, sections were incubated with primary antibodies at 4 °C overnight. Afterward, the sections were incubated with HRP-conjugated secondary antibody (Beyotime, China; A0208, 1:400) at room temperature for 1 h. Hematoxylin and dehydration were used to counterstain the nucleus. Then slides were submerged in graded ethanol and xylene and covered with coverslips. The pictures were scanned by panoramic slice scanner (Pannoramic MIDI/P250, 3DHISTECH, Hungary). The primary antibodies used were *anti*-IL-1α (Abclonal, China; A1316; 1:200), *anti*-Phospho-NRF2-S40 (Abclonal, China; AP1133; 1:200), *anti*-Phospho-NF-κB p65 (Ser536) (Abclonal, China; AP0124; 1:200), *anti*-pThr345-IRAK4 (Bioss, China; bs-10208R; 1:500), and *anti*-Ki-67 (Abcam, UK; ab16667; 1:250).

### Spatial transcriptomics data processing

Spatial transcriptomics data (GSE220978) were processed using Seurat v4.4.0 (PMID: 34062119) in R v4.3.2. Raw counts were normalized via the SCTransform function with negative binomial regression. Four samples were integrated using the merge function to correct batch effects. Differential gene expression analysis between malignant and non-malignant regions was performed with FindMarkers (log_2_ fold-change threshold: 0.25; adjusted *p* < 0.05). Spatial domains along malignant-boundary-nonmalignant axes were delineated using Cottrazm v1.1.0 (PMID: 36806082) in Python 3.8, implementing graph-based clustering with a resolution parameter of 0.8.

### Analysis of hypoxia

Hypoxia-related gene signatures were assessed using Gene Set Enrichment Analysis (GSEA). The HALLMARK_HYPOXIA gene set was obtained from the Molecular Signatures Database (MSigDB v7.5.1). Using the GSVA package (v1.50.5; Hänzelmann et al., 2013) in R, enrichment scores were calculated for all spatial spots. Within tumor regions (malignant domains confirmed by pathology annotation), spots with hypoxia scores in the top quartile were classified as Hypoxic.Tumor.Area, while the remaining tumor spots were designated Nonhypoxic.Tumor.Area.

### Bioinformatic analysis

Transcriptomic data from the TCGA-HNSC cohort were acquired via the Genomic Data Commons (GDC) portal using the TCGAbiolinks R package (v2.28.0) following standard protocols 24. GSEA was performed with clusterProfiler (v4.8.3) 25, utilizing the Hallmark gene sets from MSigDB (v2023.2; http://www.gsea-msigdb.org). Single-cell RNA sequencing data of HNSCC were processed using Seurat (v4.3.0) 26. Survival analysis and RNA expression profiling were conducted in GEPIA2 27. Protein expression patterns were validated via LinkedOmicsKB using TCGA proteomics datasets 28.

### Data visualization and statistical analysis

Data visualization was implemented with ggplot2 v3.4.4 and ggsci v3.0.0. All statistical analyses were performed in R v4.3.2. Differential gene expression between Hypoxic.Tumor.Area (top 25% hypoxia score) and Nonhypoxic.Tumor.Area was assessed using Wilcoxon rank-sum tests (Mann-Whitney U test), with statistical significance defined as *p* < 0.05 after Benjamini-Hochberg correction for multiple testing. Data are presented as mean ± SEM. Normality and homogeneity of variance were assessed using Shapiro-Wilk and Levene's tests, respectively. Statistical comparisons were performed as follows: Two-group comparisons with unpaired or paired two-tailed Student's t-tests (for parametric data); Multi-factor comparisons with Two-way ANOVA with Tukey's post hoc test (for tumor volume analysis); Survival analysis with Kaplan-Meier curves with log-rank test. All analyses were conducted in GraphPad Prism v9.5.0. Statistical significance thresholds were defined as *p* > 0.05 (ns), *p* < 0.05 (*), *p* < 0.01 (**), *p* < 0.001 (***), *p* < 0.0001 (****).

## Results

### IL-1α accumulates in hypoxic niches of HNSCC tumors

**Figure 1:**
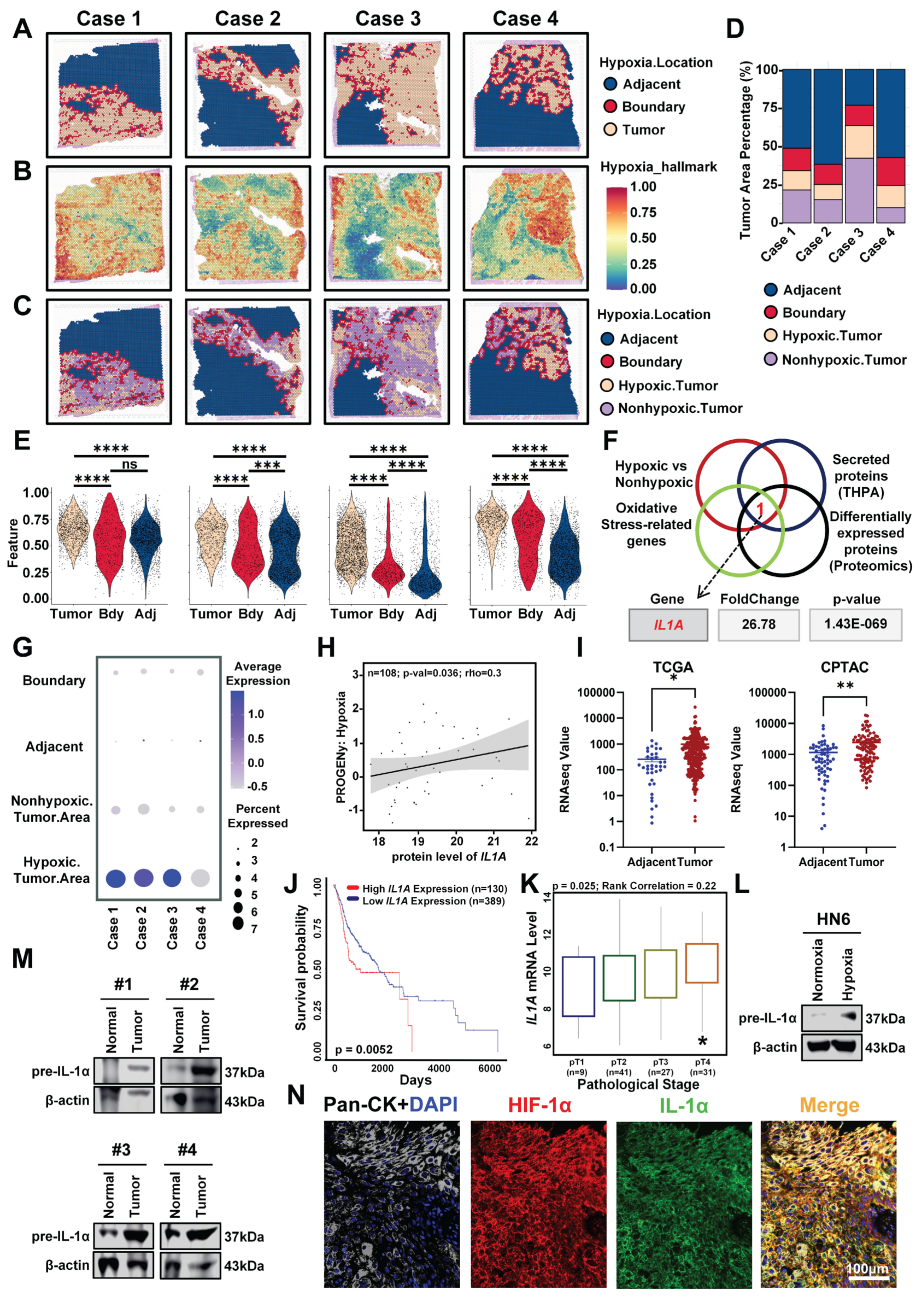
** IL-1α is highly expressed in the hypoxic area of tumor tissues of HNSCC patients. (A)** Delineation of tumor regions in four HNSCC specimens using COTTRAZM spatial transcriptomics: malignancy (yellow), boundary (red), and adjacent normal (blue).** (B)** Spatial heatmaps of hypoxia scores (HALLMARK_HYPOXIA gene set) in four HNSCC cases, demonstrating confinement of hypoxic foci within malignant regions.** (C)** Identification of hypoxic (yellow) and non-hypoxic (purple) areas within tumor regions by LGSI, illustrating sharp hypoxia-normoxia transitions.** (D)** Proportional distribution of tissue spots across regions: adjacent normal, boundary, hypoxic tumor, and non-hypoxic tumor.** (E)** Violin plots depicting hypoxia-related gene expression levels across regions (adjacent, boundary, non-hypoxic tumor, hypoxic tumor) in four cases.** (F)** Enrichment analysis of differentially expressed proteins between hypoxic and non-hypoxic tumor regions, highlighting *IL1A* among oxidative stress-related secreted factors.** (G)** Bubble plot showing spatial distribution of *IL1A* mRNA expression levels across regions (adjacent, boundary, non-hypoxic tumor, hypoxic tumor) in four samples.** (H)** Correlation analysis between *IL1A* expression and hypoxia signature scores in the TCGA HNSCC cohort.** (I)** *IL1A* mRNA expression in HNSCC tumors versus normal tissues from the TCGA (left) and CPTAC (right) databases.** (J)** Kaplan-Meier survival curves for HNSCC patients stratified by high (red) and low (blue) *IL1A* expression.** (K)** *IL1A* expression levels across pathological stages (I-IV) in the TCGA HNSCC cohort.** (L)** Western blot analysis of IL-1α protein expression in HN6 cells exposed to normoxia (21% O_2_) or hypoxia (1% O_2_).** (M)** Western blot analysis of IL-1α in paired HNSCC tumor and adjacent normal tissues from three representative patients.** (N)** Immunofluorescence co-staining of Pan-CK (white), IL-1α (green), and HIF-1α (red) in HNSCC patient tumor sections, demonstrating co-localization (yellow) in hypoxic niches. Nuclei were counterstained with DAPI (blue). Scale bars, 100 μm. ns: no significance, *p < 0.05, **p < 0.01, ***p<0.001, ****p < 0.0001.

Hypoxic heterogeneity drives PDT resistance in HNSCC, yet its spatial organization remains uncharacterized 15. To resolve this issue, the hypoxia gradients in four HNSCC specimens (GSE220978) were mapped using spatial transcriptomics with Cottrazm, which leverages single-cell RNA sequencing (scRNA-seq). We delineated malignant boundaries (Figure 1A) and mapped hypoxia via the HALLMARK_HYPOXIA gene set. Heatmap visualization confirmed hypoxia foci were confined within tumor tissues (Figure 1B), occupying nearly 50% of malignant areas (Figure 1D). Local Spatial Gradient Inference (LGSI) quantified sharp hypoxia-normoxia transitions (Figure 1C), demonstrating significant spatial heterogeneity. Crucially, hypoxia-related gene expression was consistently higher in malignant regions than in adjacent tissues across all specimens (Figure 1E). This establishes hypoxia as a spatially structured component of HNSCC tumors.

To identify molecular targets capable of overcoming PDT resistance within hypoxic niches, we performed an integrated analysis of differentially expressed genes between hypoxic and normoxic tumor regions, oxidative stress-related gene sets, secreted proteins, and our in-house proteomics data. This multi-dimensional intersection revealed *IL1A* as the only gene consistently upregulated in hypoxic regions across all datasets (Figure 1F, G). Moreover, multi-omics validation confirmed strong correlations between *IL1A* expression and hypoxia signatures (Figure 1H), with significant overexpression in HNSCC versus normal tissues in both TCGA and CPTAC cohorts (Figure 1I). Clinically, elevated *IL1A* expression predicted reduced survival probability and correlated with advanced pathological stages (Figure 1J, K). These data establish *IL1A* mRNA as a hypoxia-associated driver of oxidative resistance and poor prognosis.

Given the association of *IL1A* with hypoxia in multi-omics data, we next sought to investigate whether hypoxia directly induces IL-1α protein expression. Exposure of HN6 cells to hypoxic conditions (1% O_2_) for 32 h significantly increased IL-1α protein levels compared to normoxic controls, as demonstrated by Western blot analysis (Figure 1L). This dysregulation was confirmed in clinical specimens through immunoblotting of paired patient tissues, which revealed elevated IL-1α expression in HNSCC tumors versus matched adjacent normal tissues (Figure 1M). Critically, to establish the spatial co-localization of IL-1α within hypoxic niches in situ, we conducted immunofluorescence co-staining for IL-1α and the canonical hypoxia marker HIF-1α on HNSCC patient tumor sections. HIF-1α, the master transcriptional regulator of hypoxia response, serves as a definitive marker for identifying hypoxic regions 29. This analysis revealed pronounced spatial co-localization of IL-1α with HIF-1α-positive hypoxic regions within the tumor microenvironment (TME) (Figure 1N, S1), confirming the specific accumulation of IL-1α in hypoxic niches of human HNSCC tumors. Collectively, these results establish that hypoxia directly upregulates IL-1α expression *in vitro* while demonstrating specific accumulation of IL-1α within hypoxic niches of human HNSCC tumors *in vivo*, confirming its role as a hypoxia-responsive factor in the TME and providing the foundation for investigating its functional impact on PDT resistance.

### IL-1α drives PDT resistance of HNSCC via oxidative stress suppression

**Figure 2:**
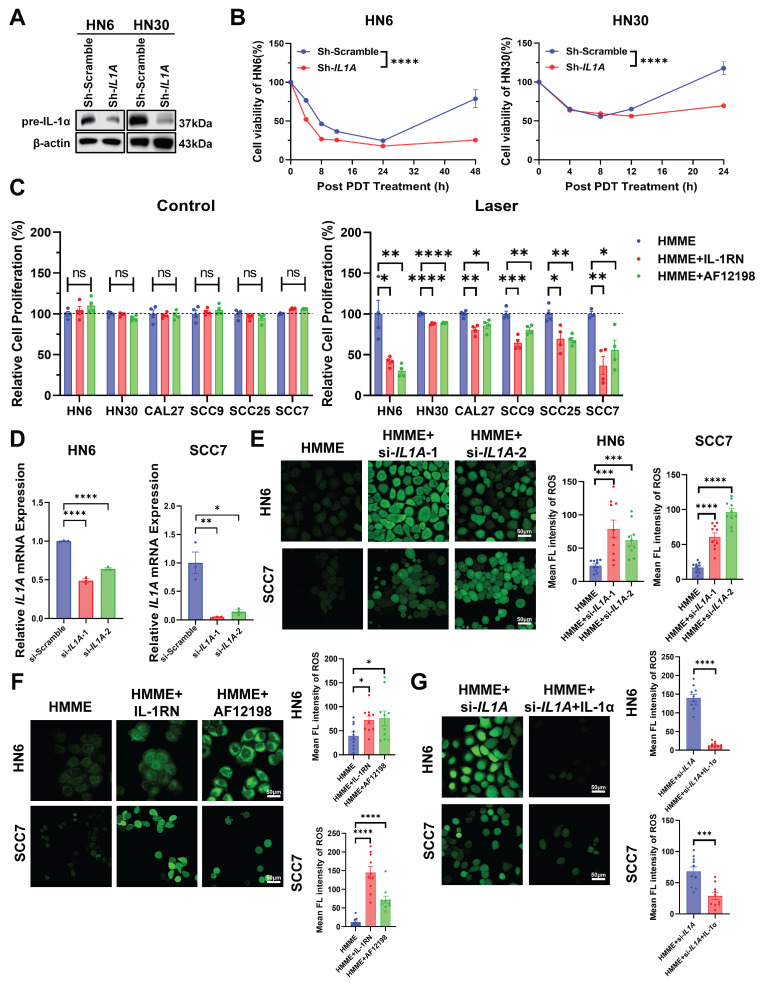
** IL-1α drives hypoxia-associated PDT resistance by suppressing oxidative stress *in vitro*. (A)** Western blot of IL-1α in hypoxic (1% O_2_) HN6/HN30 cells transduced with sh-*IL1A* or scramble shRNA.** (B)** CCK-8 assay detected the viability of sh-*IL1A* and sh-scramble HN6/HN30 cells under hypoxia after HMME-PDT at different time points.** (C)** CCK-8 assay detected the proliferation of HNSCC cells under hypoxia treated with HMME-PDT, IL-1RN, and AF12198.** (D)** *IL1A* mRNA levels in HN6 and SCC7 cells transfected with si-*IL1A* or scramble siRNA.** (E)** Representative images and statistical analysis of the mean fluorescent intensity of the ROS levels in si-*IL1A* HN6 and SCC7 cells under hypoxia after HMME-PDT. Scale bars, 50 μm.** (F)** Representative images and statistical analysis of the mean fluorescent intensity of the ROS levels in HN6 and SCC7 cells under hypoxia treated with HMME-PDT, IL-1RN, and AF12198. Scale bars, 50 μm.** (G)** Representative images and statistical analysis of the mean fluorescent intensity of the ROS levels in si-*IL1A* HN6 and SCC7 cells under hypoxia supplemented with recombinant IL-1α treated with HMME-PDT. Scale bars, 50 μm. ns: no significance, *p<0.05, **p<0.01, ***p<0.001, ****p<0.0001.

Building on the spatial enrichment of IL-1α in hypoxic niches, we functionally validated its role in PDT resistance. To determine whether IL-1α inhibition enhances PDT efficacy under hypoxia, we first established stable *IL1A* knockdown models in HN6 and HN30 cells using lentiviral shRNA. Western blot confirmed a significant reduction in IL-1α protein versus scramble controls (Figure 2A). Based on HMME uptake kinetics peaking at 3 h post-administration (Figure S2), we irradiated cells at this optimal time point, and measured the IC_50_ for HMME-PDT by CCK8 assays to determine the appropriate administration concentration and laser time (Figure S3). Under hypoxia, *IL1A* knockdown significantly enhanced PDT efficacy, reducing viability by 50% approximately versus controls in both cell lines (Figure 2B). Pharmacologic blockade of IL-1α signaling (IL-1RN and AF12198 30, 31) reduced post-PDT viability without affecting non-irradiated cells (Figure 2C), demonstrating that IL-1α specifically limits PDT cytotoxicity in hypoxic niches.

Having established IL-1α-mediated PDT protection, we investigated its role in quenching PDT-induced oxidative stress. To mechanistically dissect oxidative stress modulation while ensuring rapid and flexible genetic manipulation for acute ROS assays, we first employed transient *IL1A* knockdown using siRNA in HN6 and SCC7 cells and validated severe *reductions in IL1A* mRNA and IL-1α protein relative to scramble controls by qPCR and Western blot assays (Figure 2D, S4). Then, the intensity of green fluorescence within the cells and statistical analysis both showed that si-*IL1A* significantly amplified intracellular ROS levels in PDT-treated cells than control cells (Figure 2E). Pharmacological inhibition with IL-1RN and AF12198 also mirrored this effect, resulting in elevated intracellular ROS levels under hypoxic conditions (Figure 2F). Critically, the rescue experiments further confirmed this causal relationship. Exogenous IL-1α supplementation strongly reversed ROS accumulation in si-*IL1A* cells during PDT, reducing levels by 70% in HN6 cells and 50% in SCC7 cells compared with the non-supplement groups (Figure 2G). Similarly, the supplementation effectively attenuated the enhanced ROS fluorescence intensity observed in cells treated with IL-1RN or AF12198 (Figure S5A, B). These data demonstrate that IL-1α orchestrates an antioxidant program that blunts PDT-induced oxidative cytotoxicity in hypoxic niches.

### PDT activates NRF2 to increase IL-1α expression under hypoxic conditions

**Figure 3:**
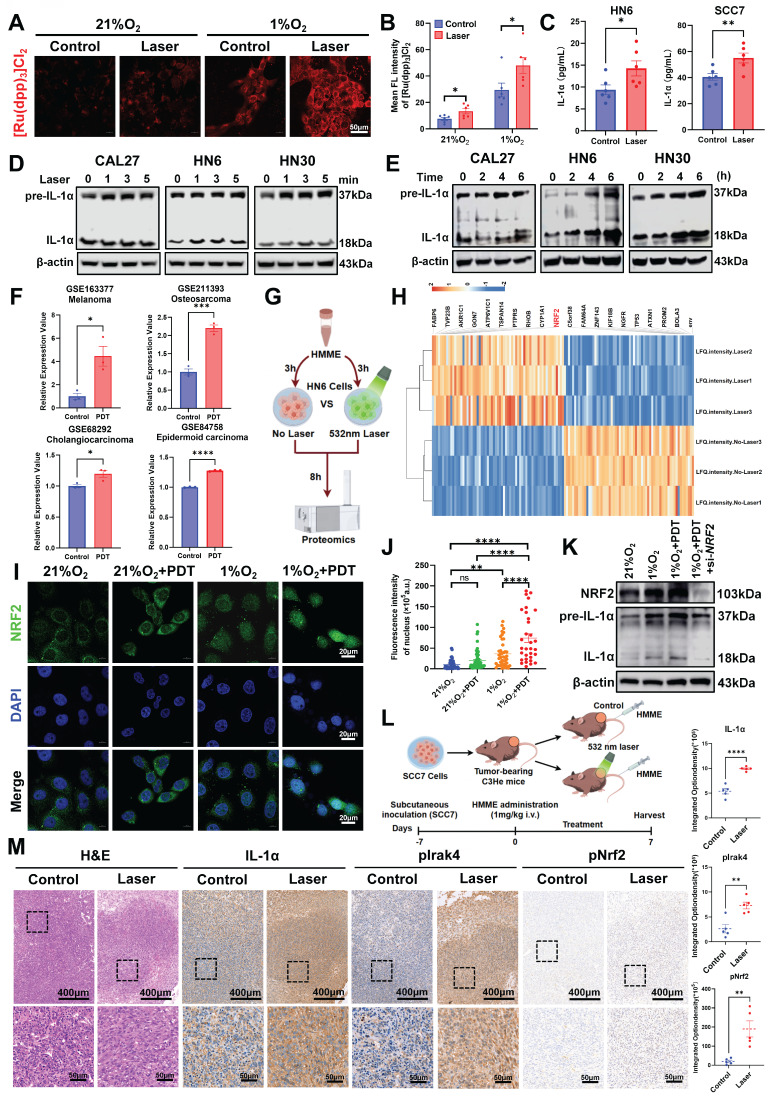
** NRF2 drives IL-1α-mediated resistance in hypoxic tumors through PDT-induced oxidative stress. (A)** Representative fluorescent images of [Ru(dpp)_3_]Cl_2_ in HN6 cells to detect intracellular oxygen under the indicated treatments in normoxic and hypoxic conditions. Scale bars, 50 μm. **(B)** Statistical analysis of mean fluorescent intensity indicated the quantification of (A). **(C)** The ELISA assay detected the expression of IL-1α in HN6 and SCC7 cells afterward PDT.** (D)** Western Blot assay detected the expression of IL-1α in HNSCC cell lines under different laser irradiation times.** (E)** Western Blot assay detected the expression of IL-1α in HNSCC cell lines under different protein collection times after PDT.** (F)** The *IL1A* mRNA expression levels in melanoma, osteosarcoma, cholangiocarcinoma, and epidermoid carcinoma after PDT.** (G)** The Schematic diagram of HN6 cells treated with HMME-mediated PDT and subjected to proteomic analysis.** (H)** Heatmap displays the up-regulation of classical antioxidant transcription factor NRF2 after HN6 cells were treated with PDT compared to the control group.** (I)** Representative images of NRF2 location as well as expression in HN6 cells under the indicated treatments in normoxic and hypoxic conditions. Scale bars, 20 μm.** (J)** Statistical analysis of mean fluorescent intensity indicated the quantification of (I).** (K)** Western Blot assay detected the expression of NRF2 and IL-1α in si-*NRF2* HN6 cells after PDT.** (L)** Schematic diagram of SCC7 cells subcutaneous implantation and the procedure of PDT.** (M)** Representative images of each group of H&E staining, IHC analysis, and statistical quantification of IL-1α, pIrak4, and pNrf2 expression levels within tumors. Scale bars, 400 μm in the enlarged image and 50 μm in the local image. ns: no significance, *p<0.05, **p<0.01, ***p<0.001, ****p<0.0001.

Since PDT-induced oxygen consumption exacerbates tumor hypoxia, a key constraint to its efficacy 17, we first quantified hypoxia dynamics post-PDT. Using a hypoxia-sensitive fluorescent probe, we observed significant oxygen depletion in PDT-treated HN6 cells under 1% O_2_, with fluorescence intensity significantly higher than non-irradiated and normoxic controls (Figure 3A, B). To determine whether PDT further directly stimulates IL-1α expression like hypoxia, we performed time-resolved analyses. ELISA revealed rapid upregulation of IL-1α in HN6 and SCC7 cells post-HMME-PDT at 6 h, compared with the non-irradiated groups (Figure 3C). Meanwhile, Western blot also demonstrated time-dependent protein induction correlating with laser duration and post-PDT recovery (Figure 3D, E). Critically, interrogation of multi-cancer GEO datasets confirmed conserved *IL1A* upregulation post-PDT across melanoma (GSE163377), osteosarcoma (GSE211393), cholangiocarcinoma (GSE68292), and epidermoid carcinoma (GSE84758) (Figure 3F), establishing this as a pan-tumor adaptive response.

Having established PDT-induced IL-1α upregulation as a conserved adaptive response, we interrogated its transcriptional mechanism, a mechanistic gap that persisted in the field. To resolve this, we deployed quantitative proteomics in hypoxic HN6 cells post-HMME-PDT versus non-irradiated controls (Figure 3G). The heatmap showed that Nuclear factor erythroid 2-related factor 2 (NRF2) emerged as a significantly up-regulated transcription factor (Figure 3H), consistent with its role as a master antioxidant regulator during oxidative stress 32, 33. To validate that PDT could further activate NRF2 in HNSCC cells, immunofluorescence assays validated PDT-induced NRF2 activation under hypoxia, showing 4-fold increased nuclear translocation in treated HN6 cells versus controls (Figure 3I, J). Functionally, *NRF2* siRNA abolished PDT-induced IL-1α upregulation under hypoxia, as confirmed by Western blot (Figure 3K). These data identify NRF2 as the direct transcriptional activator of *IL1A* during PDT, elucidating the mechanistic link between oxidative stress sensing and IL-1α-mediated resistance.

To validate the *in vivo* relevance of the Nrf2-IL-1α axis, we assessed its functional relevance in C3He mouse xenografts. After the tumor volume reached 200 mm^3^, the mice were treated with HMME (1 mg/kg) by tail vein injection and irradiated for 3 min or non-irradiated (Figure 3L). Paradoxically, PDT-treated tumors showed limited therapeutic efficacy with no significant growth inhibition versus controls of the tumor volume and weight (Figure S6B-D), despite preserved physiological status (Figure S6A). This therapeutic resistance was mechanistically instructive. Crucially, IHC analysis of these resistant tumors revealed concomitant upregulation of IL-1α and phospho-Nrf2 (pNrf2) (Figure 3M). Meanwhile, phosphorylation of Interleukin 1 receptor-associated kinase 4 at Thr345 (pIrak4), a key mediator of IL-1α receptor 1 (IL-1R1) signal transduction 34, was also significantly upregulated (Figure 3M). This finding mechanistically connects NRF2 activation to IL-1α downstream effectors. These data confirm that PDT activates the Nrf2/IL-1α/pIrak4 resistance axis within hypoxic niches, providing a molecular basis for the observed treatment failure and highlighting actionable targets for combinatorial therapy.

### IL-1α confers oxidative resistance to PDT through the NF-κB signaling pathway

**Figure 4:**
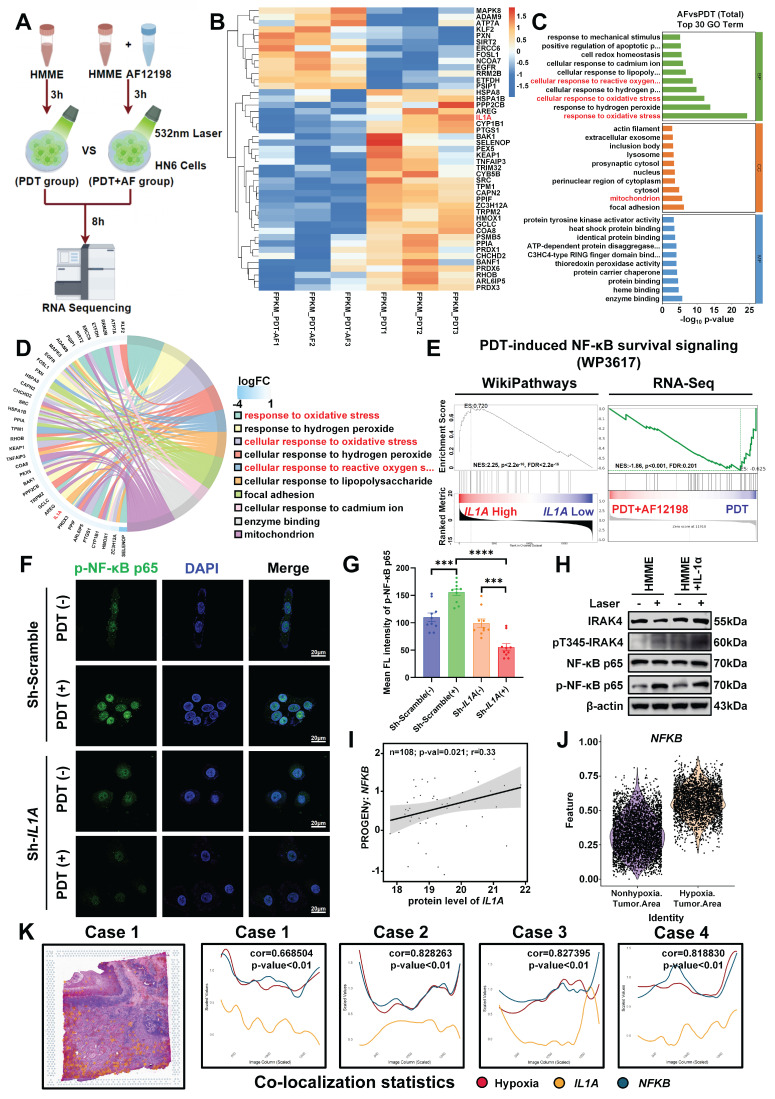
** IL-1α activates NF-κB-mediated antioxidant reprogramming to confer PDT resistance in hypoxic niches. (A)** Schematic diagram of hypoxic HN6 cells treated with IL-1R1 antagonist AF12198 post-PDT and subjected to RNA-Seq analysis.** (B)** Heatmap of downregulated oxidative stress response genes.** (C)** GO enrichment analysis investigated the cellular components, molecular functions, and biological processes associated with the differentially expressed genes.** (D)** The chord chart visualizes the pertinent signaling pathways corresponding to the enriched set of differentially expressed genes.** (E)** GSEA analysis of NF-κB pathway-related differentially expressed genes in public databases and self-measured RNA-Seq studies under PDT.** (F)** Representative immunofluorescence images of p-NF-κB p65 location in sh-*IL1A* HN30 cells under the indicated treatments. Scale bars, 20 μm.** (G)** Statistical analysis of the mean fluorescent intensity of (F).** (H)** Western blot assay on IRAK4, pIRAK4, NF-κB p65, and p-NF-κB p65 levels in the exogenously supplied IL-1α cells under HMME-PDT.** (I)** Correlation between *IL1A* mRNA levels and *NFKB* mRNA levels in HNSCC from the TCGA database.** (J)** The expression of *NFKB* in hypoxic and non-hypoxic regions of HNSCC tumor tissues.** (K)** Colocalizing hypoxia-related gene sets, *IL1A* mRNA, and *NFKB* mRNA in four cases. “+” refers to 532 nm laser irradiation, “-” refers to no irradiation. ***p < 0.001, ****p < 0.0001.

To define the antioxidant mechanism underlying IL-1α confers PDT resistance, RNA-seq of HN6 cells treated with IL-1R1 antagonist AF12198 post-PDT revealed profound suppression of oxidative stress response genes (Figure 4A, B). Enrichment analysis confirmed the differential expression primarily in oxidative stress pathways (Figure 4C, D). Among these, the NF-κB signaling pathway emerged as a key oxidative stress countermeasure, consistent with its established role in activating antioxidant genes (e.g., SOD2, HMOX1) during inflammatory stress 23, 35. Critically, GSEA analyses demonstrated significant downregulation of NF-κB signaling, aligning with *IL1A* and NF-κB pathway co-activation patterns in TCGA-HNSCC data (Figure 4E). These results establish that IL-1α activates the NF-κB signaling pathway to counteract PDT-related oxidative stress.

Functional validation established that *IL1A* knockdown abolished PDT-induced nuclear translocation of phospho-NF-κB p65 (p-NF-κB p65), reducing nuclear accumulation by >3-fold versus scrambled controls (Figure 4F, G). This phenotype was replicated by both pharmacological IL-1R1 blockade (AF12198) and genetic *IL1A* silencing (Figure S7A, B). Conversely, exogenous IL-1α enhanced IRAK4 and NF-κB phosphorylation in the hypoxic HN6 cells post HMME-PDT (Figure 4H), confirming IL-1α as the primary upstream activator of NF-κB under oxidative stress. Moreover, the expression levels of *IL1A* mRNA positively correlated with *NFKB* mRNA (Figure 4I). Spatial transcriptomics further demonstrated co-enrichment of *IL1A* and *NFKB* within hypoxic niches (Figure 4J, K), mechanistically linking microenvironmental stress sensing to transcriptional reprogramming. Collectively, these data demonstrate that PDT-induced oxidative stress triggers NRF2-mediated IL-1α expression in hypoxic microdomains, which activates IRAK4/NF-κB signaling to mitigate oxidative damage. This spatiotemporally regulated cascade drives therapeutic resistance and identifies druggable nodes for combination therapies.

### *IL1A* knockdown potentiates the antitumor efficacy of PDT *in vivo*

**Figure 5:**
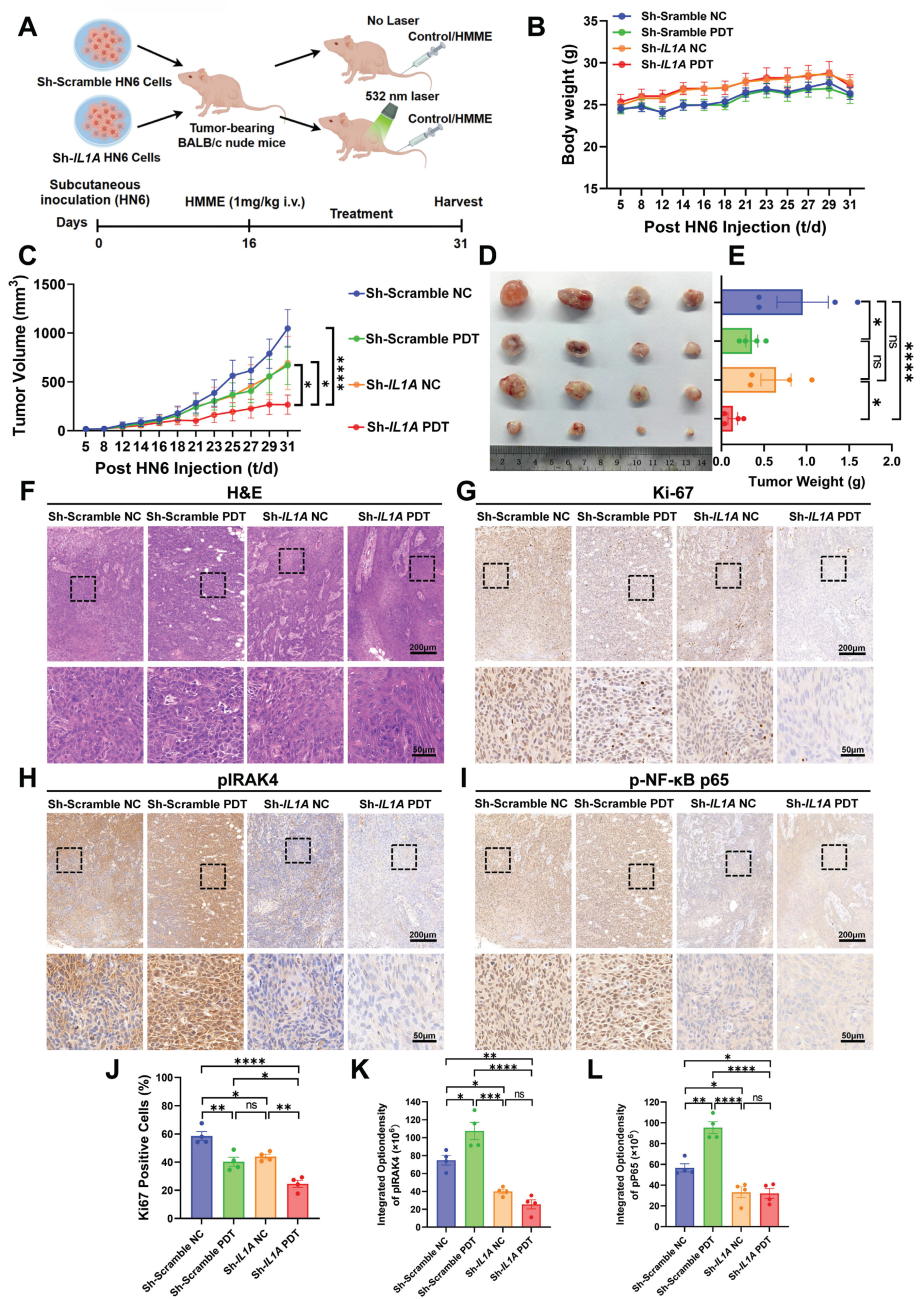
***IL1A* ablation disrupts the IL-1α/NF-κB axis to potentiate PDT efficacy *in vivo*. (A)** Schematic diagram of BALB/c nude xenografts with sh-*IL1A* HN6 cells treated with HMME-PDT (532 nm laser).** (B)** Statistical quantification of body weight at the indicated days after the mice were treated with the indicated treatment. **(C)** Tumor growth curves at the indicated days after the mice were treated with the indicated treatment.** (D)** Representative images of tumor volume at the end of treatment.** (E)** Statistical quantification of tumor weight at the end of treatment.** (F)** Representative images of each group of H&E staining of the tumor. Scale bars, 200 μm in the enlarged image and 50 μm in the local image.** (G)** Representative images of each group of Ki-67 staining of the tumor. Scale bars, 200 μm in the enlarged image and 50 μm in the local image.** (H)** Representative images of each group of pIRAK4 staining of the tumor. Scale bars, 200 μm in the enlarged image and 50 μm in the local image.** (I)** Representative images of each group of p-NF-κB p65 staining of the tumor. Scale bars, 200 μm in the enlarged image and 50 μm in the local image.** (J)** Statistical analysis of the Ki-67 positive cells indicated the quantification of (G).** (K)** Statistical analysis of the integrated density of pIRAK4 indicated the quantification of (H).** (L)** Statistical analysis of the integrated density of p-NF-κB p65 in nuclei indicated the quantification of (I). ns, no significant difference, ns: no significance, *p<0.05, **p<0.01, ***p<0.001, ****p<0.0001.

Motivated by our mechanistic delineation of the IL-1α/NF-κB antioxidant axis and *in vitro* evidence that sh-*IL1A* suppresses HNSCC proliferation post-HMME-PDT, we assessed the therapeutic impact of *IL1A* knockdown in BALB/c nude mouse xenografts. Mice implanted with sh-*IL1A* or sh-Scramble HN6 cells received HMME-PDT at tumor volumes of 20 mm^3^ (Figure 5A). No significant body weight changes indicated minimal toxicity (Figure 5B). Notably, while *IL1A* depletion and PDT alone moderately inhibited tumor growth compared with the sh-Scramble group, their combination achieved markedly enhanced suppression (Figure 5C, D). Tumor weight quantification confirmed >60% reduction in the sh-*IL1A*+PDT group versus sh-Scramble controls, whereas *IL1A* knockdown alone showed no significant decrease (Figure 5E). These data indicate that genetic ablation of *IL1A* sensitizes tumors to PDT *in vivo*, yielding synergistic antitumor efficacy without observable toxicity.

The H&E staining of tumor tissues revealed characteristic HNSCC features in all groups, as evidenced by cell arrangement, morphology, and differentiation patterns (Figure 5F). The IHC analysis of resected tumors confirmed mechanistic disruption. Ki-67⁺ proliferating cells showed moderate reduction in sh-Scramble PDT and sh-*IL1A* NC groups, with maximal suppression in the combination group (Figure 5G, J). Furthermore, sh-*IL1A* groups exhibited concomitant suppression of pIRAK4 (Figure 5H, K) and nuclear p-NF-κB p65 (Figure 5I, L) compared with the sh-Scramble groups. This directly links *in vivo* efficacy to blockade of the IL-1α signaling cascade through IRAK4 to NF-κB. Collectively, the results demonstrated that IL-1α targeting disrupts oxidative stress adaptation in hypoxic niches, potentiating PDT-mediated tumor elimination and establishing proof-of-concept for combinatorial IL-1α targeting in hypoxic HNSCC.

### IL-1R blockade synergizes with PDT in subcutaneous xenograft model

**Figure 6:**
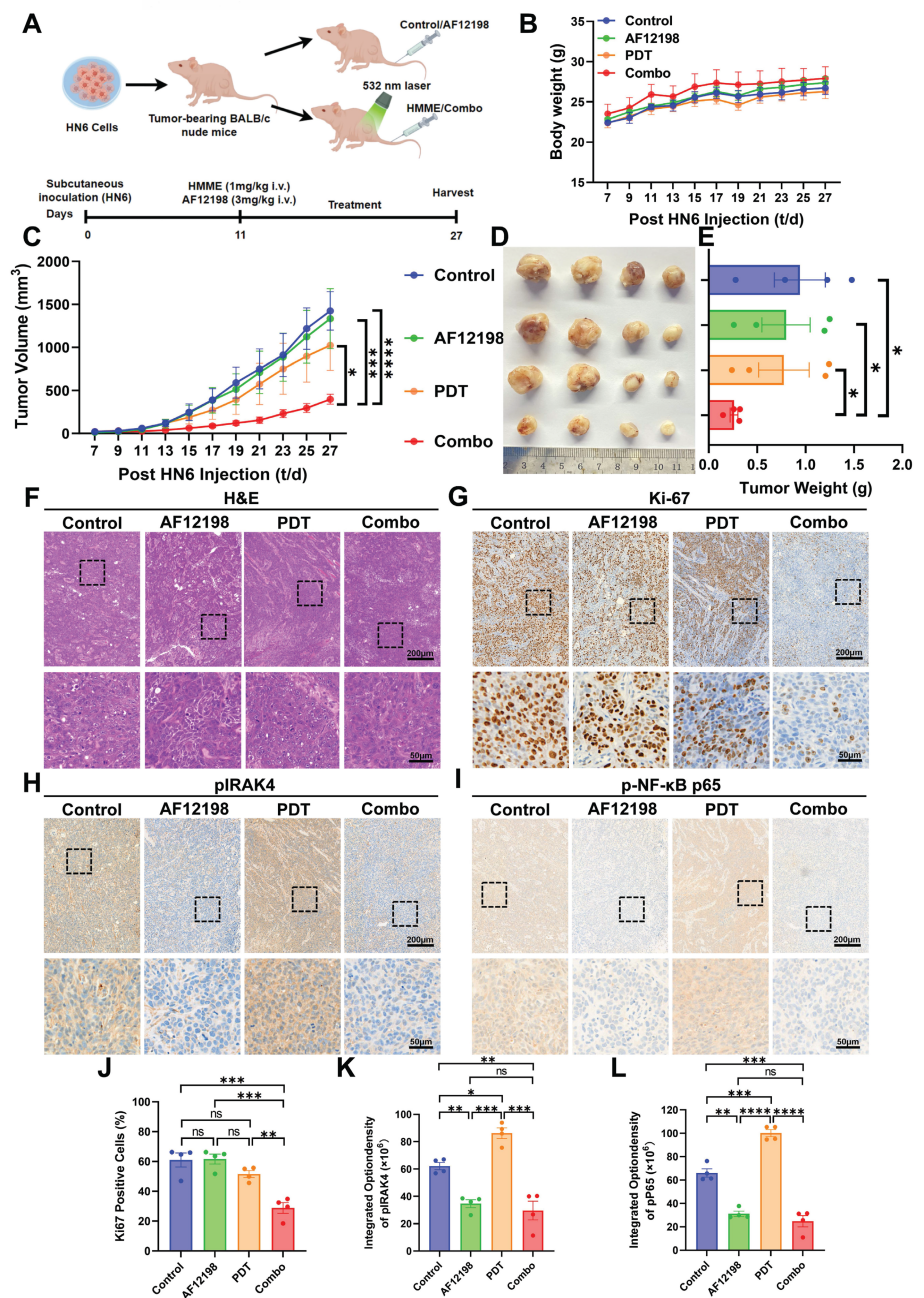
** IL-1R blockade synergizes with PDT by disrupting the IRAK4/NF-κB antioxidant axis *in vivo.* (A)** Schematic diagram of HN6 cells subcutaneous implantation and the AF12198 combining HMME-mediated PDT treatment.** (B)** Statistical quantification of body weight at the indicated days after the mice were treated with the indicated treatment.** (C)** Statistical quantification of tumor volume at the indicated days after the mice were treated with the indicated treatment.** (D)** Representative images of tumor volume at the end of treatment.** (E)** Statistical quantification of tumor weight at the end of treatment.** (F)** Representative images of each group of H&E staining of the tumor. Scale bars, 200 μm in the enlarged image and 50 μm in the local image.** (G)** Representative images of each group of Ki-67 staining of the tumor. Scale bars, 200 μm in the enlarged image and 50 μm in the local image.** (H)** Representative images of each group of pIRAK4 staining of the tumor. Scale bars, 200 μm in the enlarged image and 50 μm in the local image.** (I)** Representative images of each group of p-NF-κB p65 staining of the tumor. Scale bars, 200 μm in the enlarged image and 50 μm in the local image.** (J)** Statistical analysis of the Ki-67 positive cells indicated the quantification of (G).** (K)** Statistical analysis of the integrated density of pIRAK4 indicated the quantification of (H).** (L)** Statistical analysis of the integrated density of p-NF-κB p65 in nuclei indicated the quantification of (I). ns: no significance, *p < 0.05, **p < 0.01, ***p < 0.001, ****p < 0.0001.

Extending the therapeutic strategy beyond genetic ablation, we evaluated the IL-1R1 antagonist AF12198 in HN6 subcutaneous xenografts. After the tumor volume reached 20 mm^3^, the mice were separated into four groups: (i) vehicle control, (ii) AF12198 monotherapy, (iii) HMME-PDT monotherapy (532 nm laser for 3 min), and (iv) AF12198+PDT combination (Figure 6A). No significant body weight loss occurred during treatment, indicating stable physiological status across groups (Figure 6B). Strikingly, AF12198 synergized with PDT to drive tumor regression, performing the combination therapy achieved significant tumor growth inhibition by day 15. In contrast, the tumors treated in other groups exhibited accelerated tumor growth, especially the Control and AF12198 groups (Figure 6C). Endpoint analysis confirmed profound tumor regression in the combination group, with > 70% reduction in tumor mass versus controls (Figure 6D, E).

H&E staining confirmed typical HNSCC characteristics across all groups (Figure 6F). IHC analysis of endpoint tumors revealed maximal suppression of Ki-67⁺ proliferating cells in Combo tumors (> 2-fold reduction versus controls), whereas PDT monotherapy showed only partial proliferation reduction (Figure 6G, J). Critically, AF12198-treated groups exhibited over 2.5-fold suppression of pIRAK4 (Figure 6H, K), phenocopying genetic *IL1A* knockdown. Consistent with effective IL-1R1 blockade by AF12198, the activation of the downstream classical antioxidant factor p-NF-κB p65 was comparably suppressed in both AF12198 and Combo groups (Figure 6I, L). This pharmacological recapitulation of genetic targeting demonstrates that disrupting IL-1α signaling via either receptor antagonism or genetic ablation dismantles the IRAK4/NF-κB antioxidant axis to sensitize hypoxic HNSCC to PDT.

### IL-1R antagonism overcomes PDT resistance in PDOs of HNSCC

**Figure 7:**
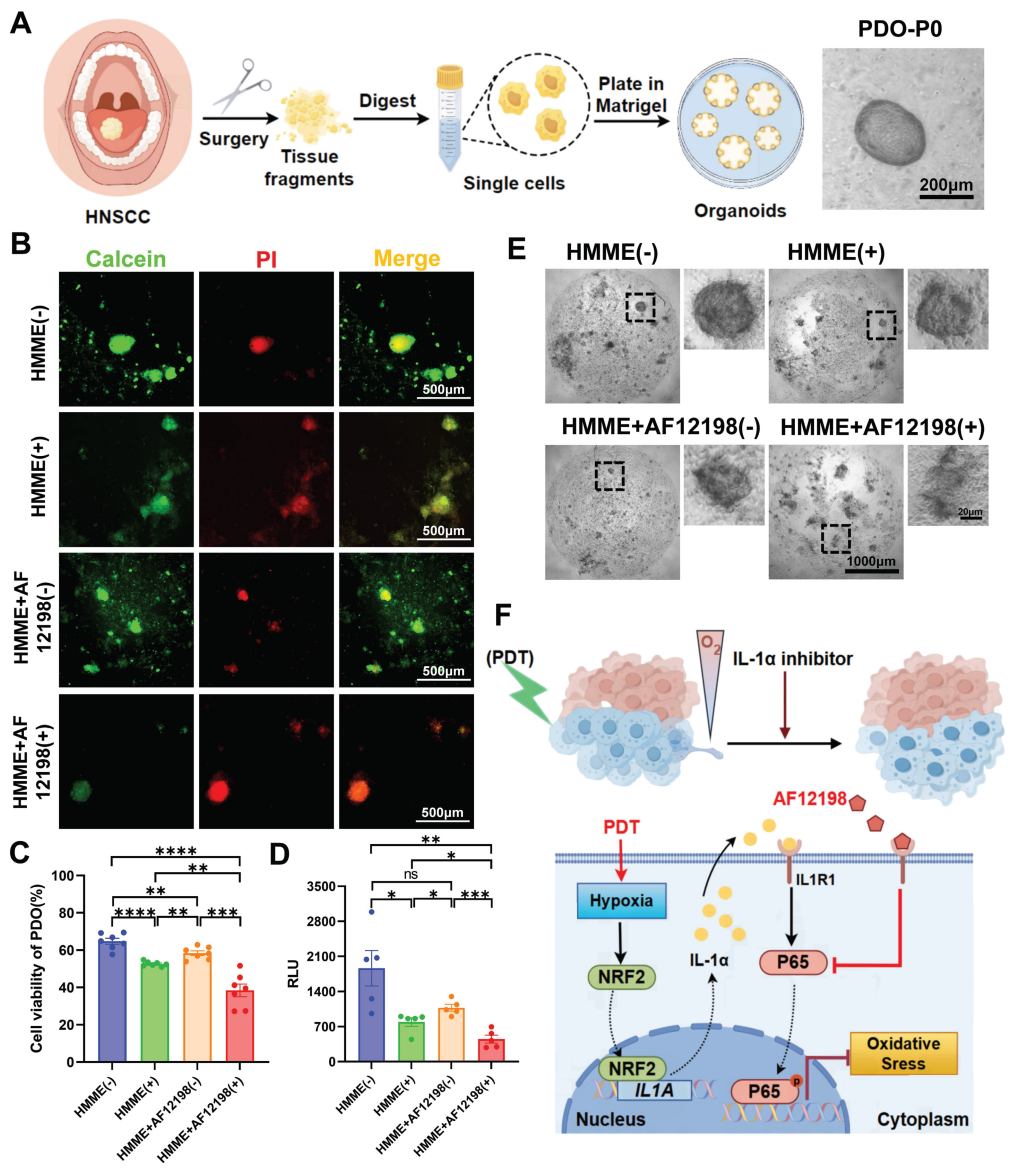
** IL-1R antagonism overcomes PDT resistance in patient-derived organoids (PDOs) of HNSCC. (A)** Schematic diagram for the establishment and culture of HNSCC PDOs from fresh surgical specimens and the representative brightfield images of PDO. Scale bars, 200 μm.** (B)** Representative live/dead fluorescence images of PDOs after 24 h treatment under four conditions. Scale bars, 500 μm.** (C)** Statistical analysis of PDOs' viability indicated the quantification of (B).** (D)** ATP levels in PDOs were measured by RLU under the indicated treatments, indicating metabolic activity.** (E)** Representative brightfield images of PDOs after 24 h treatment under four conditions. Scale bars, 1000 μm in the enlarged image and 20 μm in the local image.** (F)** Schematic representation of IL-1α promotes HNSCC resistance to PDT-induced oxidative stress under the hypoxic microenvironment. “+” refers to 532 nm laser irradiation, “-” refers to no irradiation. ns: no significance, *p < 0.05, **p < 0.01, ***p < 0.001, ****p < 0.0001.

To translate the therapeutic synergy observed in xenografts to human pathophysiology, we established PDOs from freshly resected HNSCC specimens to evaluate the IL-1α/NF-κB resistance axis (Figure 7A). PDOs were subjected to four treatment arms: (i) HMME without irradiation, (ii) HMME+532 nm laser (PDT), (iii) HMME+AF12198 without irradiation, (iv) PDT+AF12198 (Combo). Following 24 h treatment, viability was assessed by Calcein-AM/PI co-staining and quantitative live/dead fluorescence imaging (Figure 7B, C). Strikingly, Combo treatment induced the strongest cytotoxicity, reducing cell viability by > 1.5-fold versus PDT alone. This trend was corroborated by significantly lower ATP levels in Combo-treated PDOs versus the other groups (Figure 7D), alongside increased spheroid fragmentation observed by brightfield microscopy (Figure 7E). These results indicate that IL-1R1 blockade potentiates PDT efficacy in PDOs. Importantly, by validating this combination in PDOs, we provide robust preclinical evidence for rapid clinical translation of IL-1R antagonism as a PDT-sensitizing strategy.

## Discussion

In this study, we aimed to overcome intrinsic PDT resistance and limitations of conventional nanotherapies in HNSCC by identifying druggable molecular targets within therapy-resistant hypoxic niches through spatial transcriptomics and functional validation. We hypothesized that IL-1α serves as a key mediator of adaptive oxidative stress resistance in hypoxic regions, with its blockade restoring PDT efficacy. Our results confirmed that IL-1α is robustly upregulated in hypoxic zones of human HNSCC and further induced by PDT through NRF2. Mechanistically, IL-1α binding to IL-1R1 triggers a downstream IRAK4/NF-κB signaling cascade that enhances antioxidant defense and attenuates ROS-mediated cytotoxicity. Genetic knockdown and pharmacological inhibition of IL-1α or IL-1R1 significantly sensitized hypoxic tumor cells and PDOs to PDT, synergistically reducing viability and tumor growth across preclinical models. Collectively, these findings demonstrate that targeting the IL-1α/IL-1R1/NF-κB axis effectively disrupts a central adaptive resistance pathway, offering a clinically actionable strategy to enhance PDT outcomes in HNSCC (Figure 7F).

Our findings establish the IL-1α/IL-1R1/NF-κB axis as a previously underappreciated and hypoxia-induced signaling cascade that confers robust resistance to oxidative stress during PDT in HNSCC. Spatially heterogeneous tumor microenvironments drive treatment failure through hypoxic gradients that promote metabolic reprogramming, immunosuppression, and therapy resistance 36. Critically, clinically deployed HMME-PDT exacerbates intratumoral hypoxia due to its oxygen-consuming nature, creating a self-perpetuating cycle that limits ROS generation and enhances antioxidant adaptation 10. Although IL-1α has been implicated in tumorigenesis and oxidative stress adaptation 37, 38, such as through vascular endothelial growth factor (VEGF)-mediated regeneration post-PDT 39 and glutathione synthesis under nutrient deprivation in HNSCC 32, its spatial coordination of PDT resistance in hypoxic niches was previously uncharacterized. Therefore, spatial transcriptomics revealed pronounced co-localization of IL-1α with HIF-1α-enriched regions, identifying it as a microenvironmentally-driven factor amplified by chronic hypoxia and acute PDT stress. Mechanistically, PDT-induced oxidative stress triggers NRF2-mediated IL-1α transcription, activating IL-1R1/IRAK4/NF-κB signaling to initiate an antioxidant transcriptional program. This pathway represents a coordinated adaptive response that substantially attenuates ROS-mediated cytotoxicity.

Despite its advantages in safety and tolerability, PDT efficacy is fundamentally constrained by hypoxia-driven resistance 1, 9. The therapeutic effect relies critically on ROS generation to induce lethal photodamage 40. However, the hypoxic tumor microenvironment activates HIF-1α signaling during PDT, promoting cell-protective autophagy and enhancing antioxidant defenses. Such as the key adaptations GPx4 upregulation that suppresses lipid peroxidation 22, 41, fostering treatment-resistant cell populations with >3-fold greater PDT resistance versus normoxic cells 42, 43. Consequently, numerous studies have focused on developing novel nano-photosensitizers to alleviate tumor hypoxia 17. Yet nanotechnology-based strategies face considerable translational challenges, including prolonged development timelines, high production costs, and unintended activation of compensatory signaling pathways that sustain treatment resistance and diminish PDT efficiency 44. In contrast, our work identifies a targetable cell-signaling node within the hypoxic niche. Using the clinically deployed HMME (FDA-approved for port-wine stain PDT), we demonstrate that AF12198-mediated IL-1α/IL-1R1 blockade disrupts hypoxia-adaptive signaling and rescues PDT efficacy across models without complex nanomaterials. This work fundamentally advances our understanding of the dynamic interplay between tumor microenvironmental stress and therapy resistance by revealing how a hypoxic-immune signal orchestrates transcriptional antioxidant programs to protect cells from PDT cytotoxicity.

Looking forward, clinical translation of IL-1α blockade warrants further investigation, though current study limitations require acknowledgment. First, while our models (cell lines, xenografts, and PDOs) collectively support the IL-1α/NF-κB axis's role in PDT resistance, these systems cannot fully replicate human tumors' immune context and stromal complexity. The absence of intact immunity may underestimate the full IL-1α blockade's therapeutic potential, particularly regarding immunomodulatory effects. Accumulating evidence indicates that IL-1α not only modulates immunosuppressive myeloid cells and inhibits T-cell activation but also can be augmented under serum deprivation and hypoxic stress to promote the immune-suppressive activity of mesenchymal stromal cells in OSCC models 38, 45. Thus, future studies should explore whether combining IL-1α blockade with PDT-induced immunogenic cell death can remodel the tumor microenvironment, activate antitumor immunity, and ultimately reduce metastatic progression. Second, although AF12198 showed significant synergy with PDT, its selectivity and off-target effects require comprehensive characterization. Conversely, the IL-1α monoclonal antibody directly acts on IL-1α itself, potentially exhibiting high specificity and a superior inhibitory effect. Therefore, the use of clinical-grade monoclonal antibodies against IL-1α or IL-1R1, such as Bermekimab (NMPA Approval) 46, Rilonacept (FDA Approval) 47, and Anakinra (FDA Approval) 48, would better clarify the pathway contributions and enhance translational relevance. Third, the PDO sample size was relatively small despite demonstrating robust combinatorial effects. Validation across larger multi-center PDO cohorts representing diverse HNSCC subtypes and genetic backgrounds remains essential. Finally, although NRF2 regulates IL-1α induction, other hypoxia-sensitive transcription factors may contribute, and their roles need further dissection.

Notably, these limitations do not compromise our core conclusions. The consistency of responses across genetic and pharmacological perturbation *in vitro* and *in vivo*, reinforced by spatial transcriptomic evidence from human tumors, robustly supports the functional relevance of the IL-1α/NF-κB pathway in PDT resistance. Rescue experiments with exogenous IL-1α and pathway inhibition specificity demonstrated through multiple antagonists further validate our mechanistic claims. Future study using immune-competent murine models, clinical-grade IL-1α blocking antibodies, and expanded PDO platforms will address these gaps and accelerate the clinical translation of this combination strategy.

In summary, our study identifies the IL-1α/IL-1R1/NF-κB axis as a therapeutically actionable pathway driving PDT resistance in hypoxic tumor niches. By combining spatial transcriptomics with functional validation in patient-derived models, we demonstrate that IL-1R blockade disrupts adaptive antioxidant responses and synergizes with PDT, providing a readily translatable alternative to complex nanomaterial-based approaches. This work not only deciphers microenvironment-driven resistance mechanisms but also establishes a clinically feasible approach to enhance PDT efficacy in HNSCC and potentially other solid tumors.

## Supplementary Material

Supplementary figures.

## Data Availability

All data supporting the findings of this study are available within the paper and its Supplementary Information.
