# Supplementary material for: Targeting IL-1α Sensitizes HNSCC to PDT by Reversing Hypoxia and NF-κB-Driven Oxidative Stress Resistance
Source: Int J Biol Sci. 2026 Jun 4;22(11):5988–6008. doi: 10.7150/ijbs.131696 (PMC13282802; doi:10.7150/ijbs.131696)
Supplement: Supplementary file 1 — Supplementary figures. [file ijbsv22p5988s1.pdf]

Targeting IL-1α Sensitizes HNSCC to PDT by Reversing Hypoxia and NF-κB-Driven Oxidative Stress Resistance

Zhiyin Li[1,#], Yikang Ji [2,#], Xinran Zhao[2,#], Hexin Ma[2], Wanling Chen[3], Zijie Zhou[2], Xu Wang[2,*], Lingyue Shen[2,4,*], Lingyan Zheng[1,*]

1. Department of Oral Surgery, Shanghai Ninth People's Hospital, Shanghai Jiao Tong University, School of Medicine; College of Stomatology, Shanghai Jiao Tong University; National Center for Stomatology; National Clinical Research Center for Oral Diseases; Shanghai Key Laboratory of Stomatology; Shanghai Research Institute of Stomatology; Shanghai 200011, P.R. China
2. Department of Oral and Maxillofacial-Head and Neck Oncology, Shanghai Ninth People's Hospital, Shanghai Jiao Tong University School of Medicine; College of Stomatology, Shanghai Jiao Tong University; National Center for Stomatology; National Clinical Research Center for Oral Diseases; Shanghai Key Laboratory of Stomatology; Shanghai Research Institute of Stomatology; Shanghai Center of Head and Neck Oncology Clinical and Translational Science; Shanghai 200011, P.R. China
3. Department of Oral Pathology, Shanghai Ninth People's Hospital, Shanghai Jiao Tong University, School of Medicine; College of Stomatology, Shanghai Jiao Tong University; National Center for Stomatology; National Clinical Research Center for Oral Diseases; Shanghai Key Laboratory of Stomatology; Shanghai 200011, P.R. China
4. Department of Laser and Aesthetic Medicine, Shanghai Ninth People's Hospital, Shanghai Jiao Tong University School of Medicine, Shanghai 200011, P.R. China

# Authors contributed equally to this work.

* Corresponding authors: Prof. Lingyan Zheng. Email: zhenglingyan73@163.com. Prof. Lingyue Shen. Email: SHENLY1703@sh9hospital.org.cn. Prof. Xu Wang. Email: wangx312016@sh9hospital.org.cn.

Supplementary figures

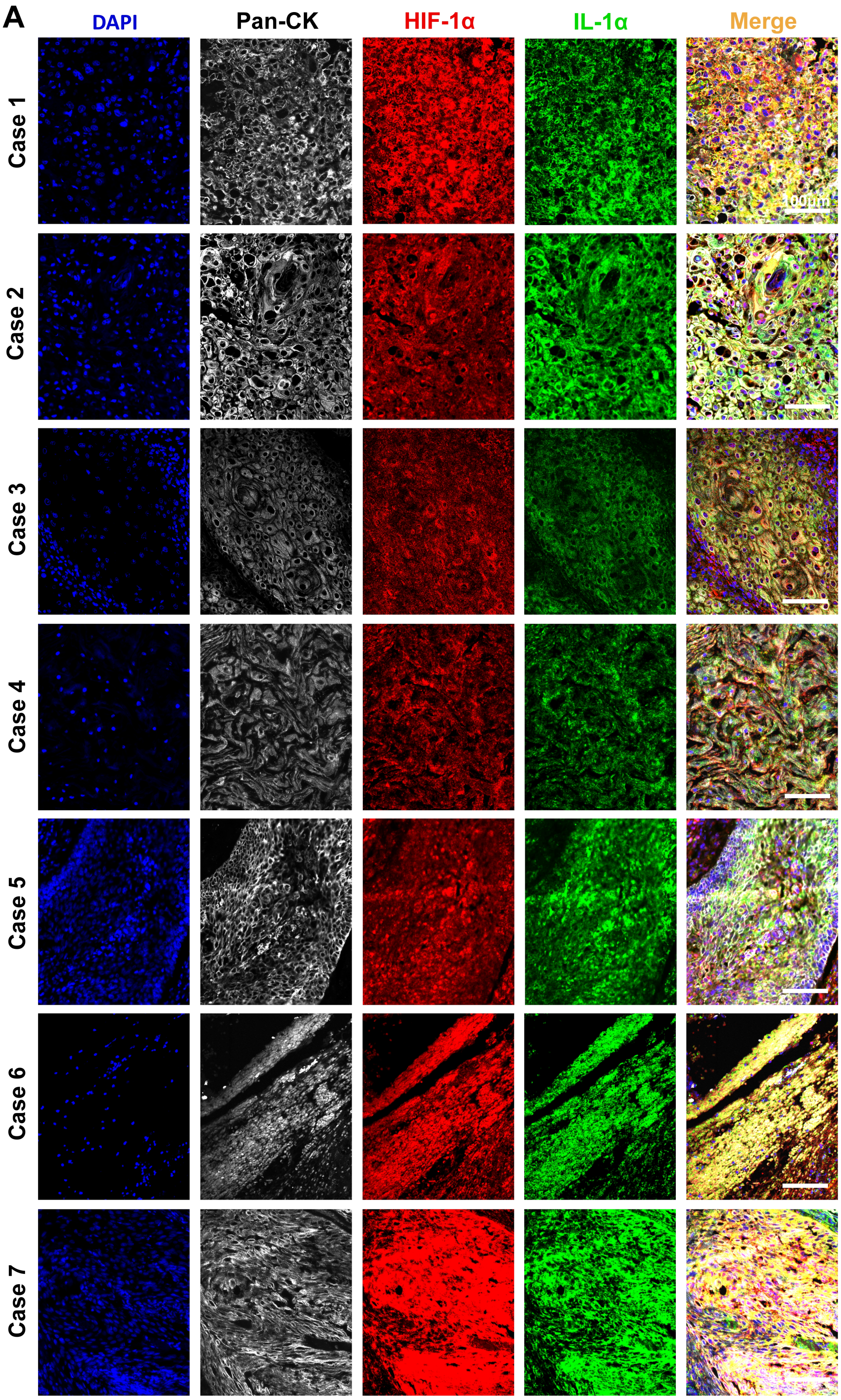

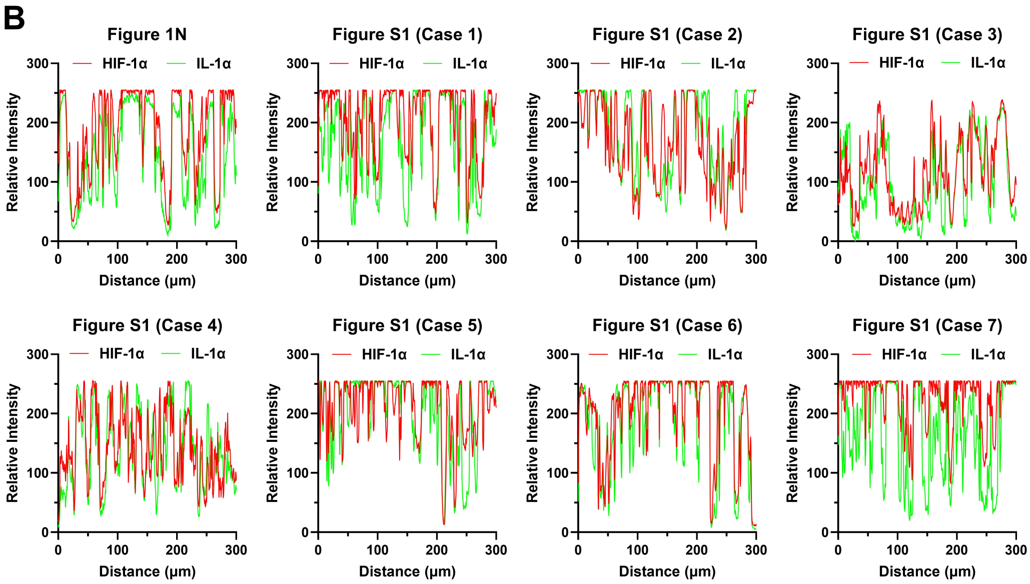

Figure S1. Immunofluorescence co-staining of Pan-CK (white), IL-1α (green), and HIF-1α (red) in HNSCC patient tumor sections, demonstrating co-localization (yellow) in hypoxic niches.

(A) Representative fluorescent images of the HNSCC patient tumor sections. Nuclei were counterstained with DAPI (blue). Scale bars, 100 μm.

(B) *The co-localization statistical analysis of HIF-1α and IL-1α in Figure 1N and S1A.*

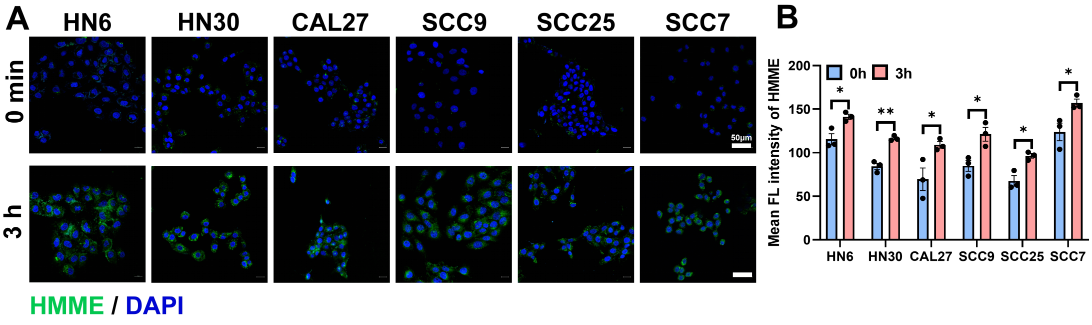

Figure S2. Intracellular uptake of HMME in HNSCC cell lines.

(A) Representative fluorescent images of HNSCC cell lines incubated with HMME for 0 min and 3 h. Scale bars, 50 μm.

(B) Statistical analysis of mean fluorescent intensity indicated the quantification of (A), **p*<0.05, ***p*<0.01.

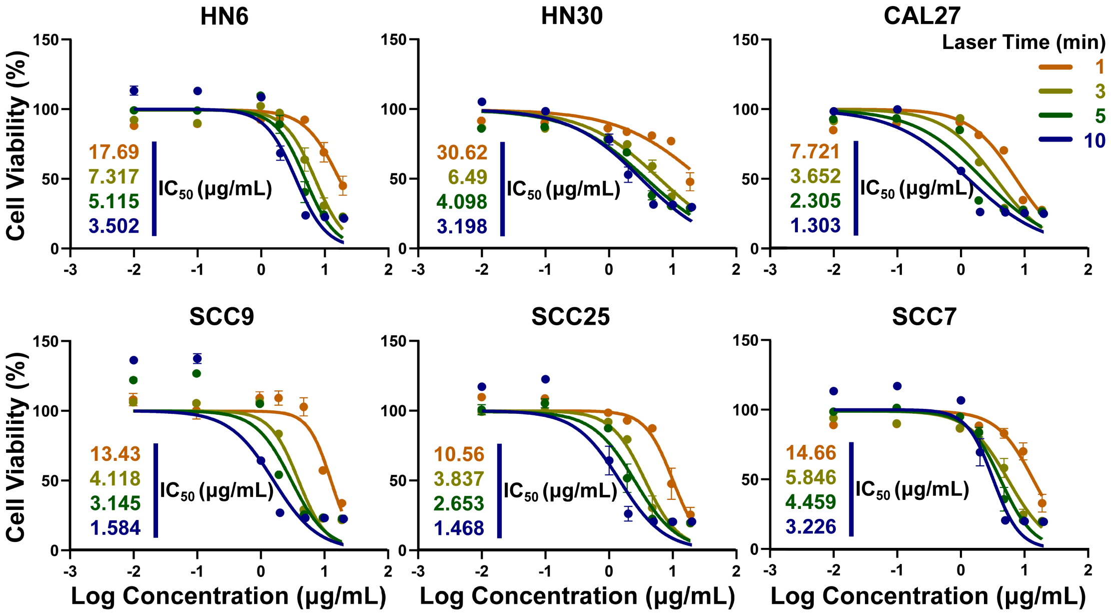

Figure S3. CKK8 assay detected HNSCC cell lines' viability and IC_{50} under the HMME-mediated PDT at different concentrations and laser time.

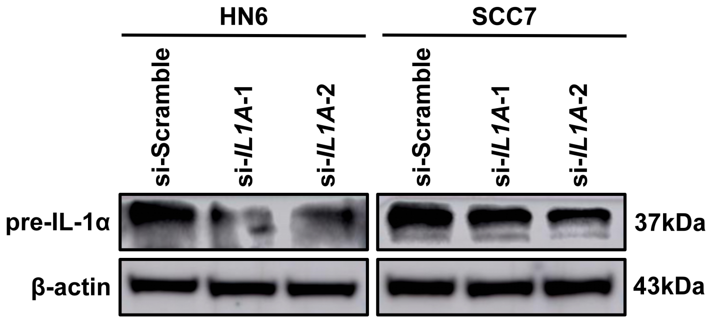

Figure S4. Western blot of IL-1α levels in HN6 and SCC7 cells transfected with si-*IL1A* or scramble siRNA.

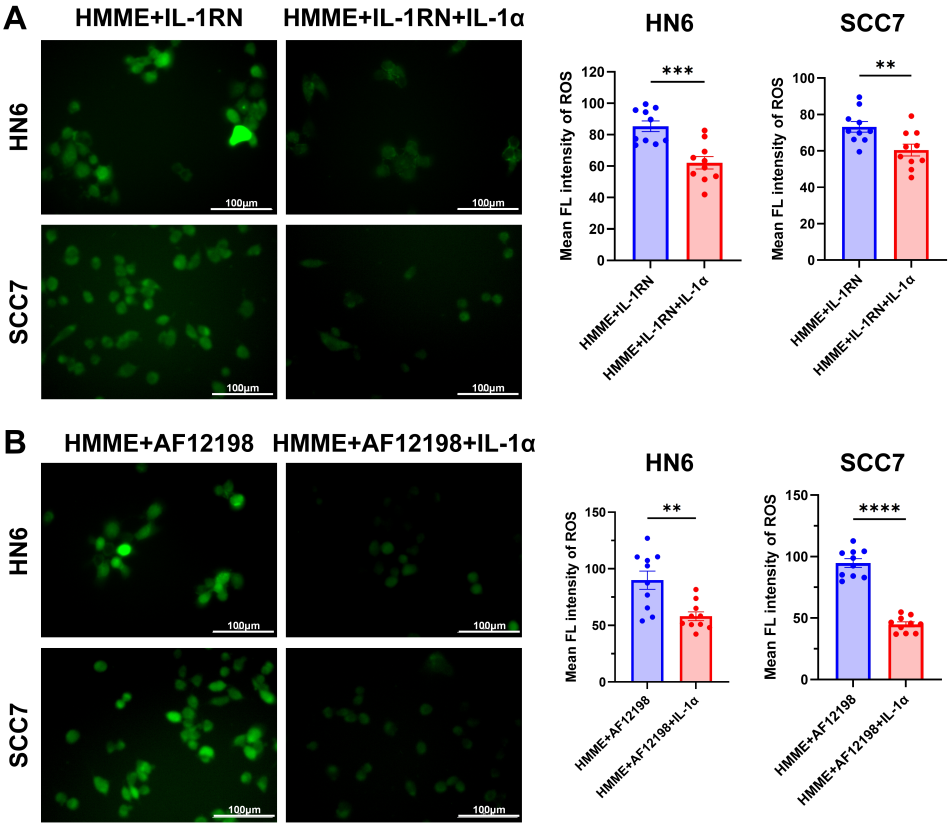

Figure S5. Representative images and statistical analysis of the mean fluorescent intensity of the ROS levels in HN6 and SCC7 cells under the indicated treatments.

(A) Representative images and statistical analysis of ROS mean fluorescence intensity in HN6 and SCC7 cells following HMME-PDT treatment, with pharmacological inhibition by IL-1RN and/or supplementation with recombinant IL-1α. Scale bars, 100 μm.

(B) Representative images and statistical analysis of ROS mean fluorescence intensity in HN6 and SCC7 cells following HMME-PDT treatment, with pharmacological inhibition by AF12198 and/or supplementation with recombinant IL-1α. Scale bars, 100 μm. $^{**}p<0.01$, $^{***}p<0.001$, $^{****}p<0.0001$.

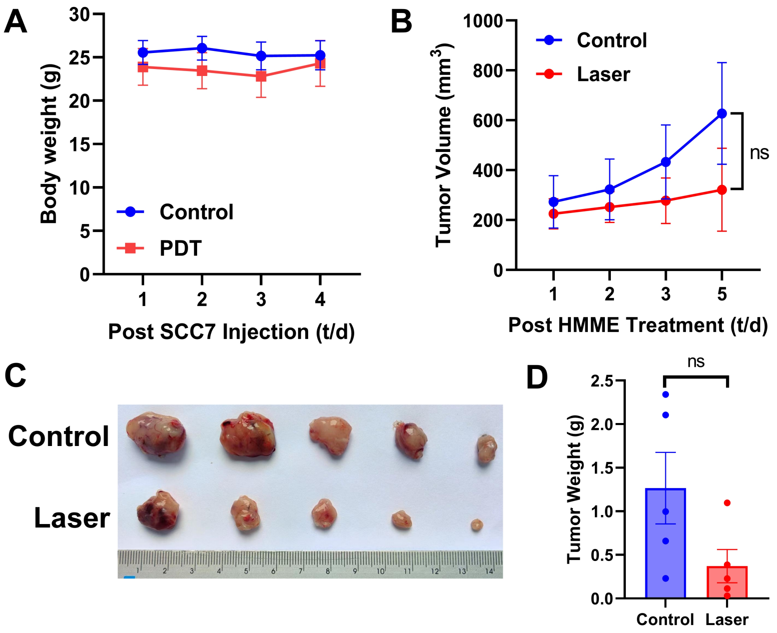

Figure S6. The therapeutic effect of HMME-mediated PDT on C3He subcutaneous tumor-bearing mice.

(A) Statistical quantification of body weight at the indicated days of the C3He xenograft models.

(B) Statistical quantification of tumor volume at the indicated days of the C3He xenograft models.

(C) Representative images of tumors in the C3He xenograft models with indicated treatments.

(D) Statistical quantification of tumor weight at the end of treatment. ns: no significance.

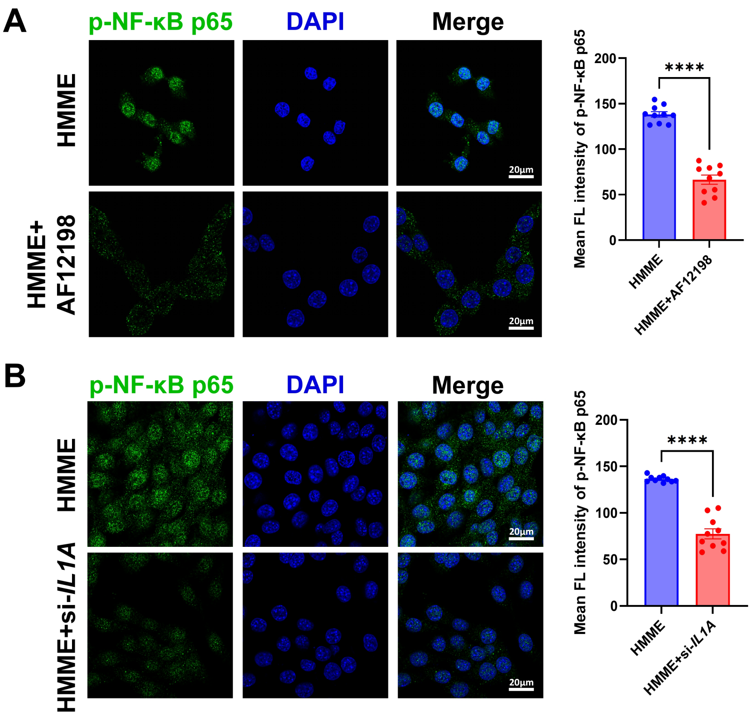

Figure S7. Representative immunofluorescence images of p-NF-κB p65 location under the indicated treatments.

(A) Representative immunofluorescence images and statistical analysis of the mean fluorescent intensity of p-NF-κB p65 location in HN6 cells treated with AF12198 and HMME-PDT. Scale bars, 20 μm.

(B) Representative immunofluorescence images and statistical analysis of the mean fluorescent intensity of p-NF-κB p65 location in si-*IL1A* HN6 cells treated with HMME-PDT. Scale bars, 20 μm. ****p<0.0001.

Supplementary Table 1 (siRNA sequences)

**SiRNA name**	**Oligonucleotides**
IL1A siRNA-1	GUCUUAAUAUUGAAAAUGACA tt,<br>UUCGUAUUUGAUGAUCCUC tt
IL1A siRNA-2	CAUCAAAGGAUGAUGCUAA tt,<br>UUAGCAUCAUCCUUUGAUG tt
NRF2 siRNA	UCAUUUUCAAUAUUAAGACAC tt,<br>GUCUUAAUAUUGAAAAUGACA tt

Supplementary Table 2 (shRNA sequences)

**SiRNA name**	**Oligonucleotides**
IL1A shRNA	CATCAAAGGATGATGCTAA tt

Supplementary Table 3 (qPCR primer sequences)

**Primer name**	**Forward sequence**	**Reverse sequence**
IL1A	TGGTAGTAGCAACCAACGGGA	ACTTTGATTGAGGGCGTCATTC
